# Posterior reversible encephalopathy syndrome across 22 VEGF-pathway inhibitors in the FDA adverse event reporting system: reporting patterns and reporting-interval characteristics

**DOI:** 10.3389/fphar.2026.1887454

**Published:** 2026-07-09

**Authors:** Ji Li, Yan Cheng, Yunzhou Yang

**Affiliations:** Department of Neurology, Lu’an People’s Hospital (The Affiliated Lu’an Hospital of Anhui Medical University), Lu’an, Anhui, China

**Keywords:** disproportionality analysis, FAERS, fruquintinib, pharmacovigilance, posterior reversible encephalopathy syndrome, ramucirumab, tivozanib, VEGFR inhibitors

## Abstract

**Objectives:**

Posterior reversible encephalopathy syndrome (PRES) is a rare but potentially severe complication of VEGF-pathway inhibition. A recent FAERS analysis supported PRES as a probable class effect of angiogenesis inhibitors but found no association with continuous VEGFR affinity, did not formally test a categorical VEGFR-2 selectivity contrast, and could not characterise time to onset. Complementing that work, we compared disproportionality signals across 22 VEGFi/VEGFRi agents and examined whether signal ranking tracked VEGFR-2 selectivity, systemic exposure, and published case-report volume.

**Methods:**

In this observational pharmacovigilance study we analysed FAERS reports (2010-Q1 to 2026-Q1) via the openFDA API. PRES was ascertained by MedDRA Preferred Terms. Four disproportionality metrics (ROR, PRR, BCPNN-IC with IC025, EBGM) were computed for each agent; robust signals were confirmed by Bonferroni and Benjamini-Hochberg correction. We characterised the drug-start-to-FDA-receipt reporting interval (a reporting-delay measure, not true clinical time-to-onset), explored the correlation between published VEGFR-2 IC50 and log-ROR, and performed a targeted PubMed scan against the case-report literature.

**Results:**

Under formal multiplicity correction (Bonferroni and Benjamini-Hochberg), 13 of 16 evaluable agents produced robust elevated PRES signals (ROR up to 16.68). Signal intensity separated cleanly by VEGFR-2 selectivity: every highly selective VEGFR-TKI (tivozanib, lenvatinib, fruquintinib, axitinib; ROR 9.74–16.68) ranked above every multi-kinase agent (ROR 2.00–6.05) (Mann-Whitney p = 0.005; Spearman rho = 0.84, p = 0.001). By contrast, the continuous IC50-ROR correlation was non-significant primarily (rho = −0.38, p = 0.25) and exclusion-sensitive, hence hypothesis-generating only. Intravitreal anti-VEGF agents produced no positive signal, an internal negative control. Nintedanib yielded an inverse signal (ROR 0.34), likely indication channelling. Tivozanib and ramucirumab (ROR 7.56) were under-represented in the PubMed case-report literature (FAERS-PubMed rho = 0.09, p = 0.78).

**Conclusion:**

PRES disproportionality varied substantially across VEGFi/VEGFRi agents, with intravitreal agents serving as an internal negative control consistent with a systemic-exposure mechanism. IC50-based analyses were small-sample and hypothesis-generating only. Tivozanib and ramucirumab emerged as under-represented signals meriting confirmation; selective VEGFR-TKI recipients may warrant close blood-pressure monitoring and a low threshold for neuroimaging.

## 1 Introduction

Posterior reversible encephalopathy syndrome (PRES) is a clinico-radiological syndrome characterised by the acute onset of headache, altered mental status, visual disturbance, and seizures, associated with reversible vasogenic oedema predominating in the parieto-occipital cortico-subcortical white matter (Hinchey et al., 1996; Fugate and Rabinstein, 2015). First described in 1996, PRES is now recognised across a heterogeneous set of clinical contexts including severe or fluctuating hypertension, eclampsia, autoimmune disease, renal failure, solid-organ and haematopoietic-stem-cell transplantation with calcineurin-inhibitor exposure, and a growing number of anti-neoplastic therapies. When recognised promptly and the offending precipitant is removed, the neurological and radiological changes reverse within days to weeks; delayed recognition, however, can result in cytotoxic infarction, haemorrhage, and permanent deficit. Drug-induced PRES is therefore a clinically actionable diagnosis, and accurate attribution at the drug level has direct implications for both patient management and future prescribing.

The vascular endothelial growth factor (VEGF) signalling pathway is a constitutive regulator of endothelial homeostasis: VEGF-A, acting through VEGFR-2, drives PI3K–Akt–eNOS activation, nitric oxide–dependent vasodilation, and blood–brain barrier integrity (Ferrara et al., 2003; Eremina et al., 2008). Pharmacological interruption of this axis is the shared mechanism of a broad and clinically heterogeneous drug class that now spans 22 agents in routine use, comprising six monoclonal antibodies or decoy receptors (bevacizumab, ramucirumab, aflibercept, ranibizumab, brolucizumab, conbercept) and sixteen small-molecule VEGFR tyrosine kinase inhibitors (TKIs). These agents span a wide range of kinome selectivity, target potency, routes of administration, and licensed indications, from metastatic solid tumours and thyroid cancer to idiopathic pulmonary fibrosis and neovascular age-related macular degeneration. Class-wide hypertension is well-documented—affecting up to 40% of patients receiving selective VEGFR-TKIs—and provides a plausible mechanistic substrate for PRES through the same endothelial-autoregulatory axis.

That a class-level association between VEGF-pathway inhibition and PRES exists has been established by case reports (Glusker et al., 2006; Martín et al., 2007), focused single-centre series (Seet and Rabinstein, 2012), narrative reviews (Tlemsani et al., 2011), and, more recently, global pharmacovigilance databases. Balcerac et al. (2023) identified a drug-level PRES signal for multiple VEGFi/VEGFRi in VigiBase, and Lei et al. (2024) produced a complementary FAERS-wide ranking; Xu et al. (2025) subsequently described the single-drug safety profile of fruquintinib. Most directly, Donati et al. (2026) recently analysed 15 anti-VEGF/VEGFR agents for PRES in FAERS and concluded that PRES is probably a class effect, integrating receptor-affinity data from ChEMBL and IUPHAR. Notably, however, their continuous receptor-affinity regression did not reveal an association with VEGFR (only an inverse FGFR4 signal), they did not formally test a categorical VEGFR-2 selectivity contrast, and they reported that missing event-date data precluded any time-to-onset analysis. A methodological antecedent for a pharmacologically constrained within-class comparator set is the recently published 22-drug within-class hypertension analysis by Kuang et al. (2024). Against this background, three clinically important questions remain incompletely resolved. First, whether PRES disproportionality signal intensity across VEGFi/VEGFRi separates by VEGFR-2 selectivity when tested categorically, given the null continuous-affinity result of Donati et al. Second, whether route of administration and licensed indication modify the signal in ways consistent with a systemic-exposure mechanism. Third, whether the disproportionality ranking matches the published case-report record—and if not, which agents are missing from the literature and should be prioritised for confirmatory description.

To address these questions, we conducted a within-class disproportionality analysis of 22 VEGFi/VEGFRi agents in the FDA Adverse Event Reporting System (FAERS, 2010-Q1 to 2026-Q1). Four prespecified hypotheses structured the analysis: (H1) that systemic VEGFi/VEGFRi as a class are associated with PRES disproportionality; (H2) that signal intensity follows a gradient of VEGFR-2 selectivity, testable both categorically and through continuous correlation with published biochemical IC_50_; (H3) that the drug-start–to–FDA-receipt interval (reporting interval) varies systematically by drug class; and (H4) that route of administration and licensed indication modify the signal, with intravitreal agents serving as an internal negative control and nintedanib as an indication-channelling test case. A secondary, targeted PubMed scan was used to cross-validate the FAERS ranking against the published case-report literature and to identify agents with strong disproportionality signals but absent literature anchors.

## 2 Materials and methods

### 2.1 Data source

This observational pharmacovigilance study analysed the FDA Adverse Event Reporting System (FAERS) public release dataset accessed via the openFDA API (https://open.fda.gov), covering the period from 1 January 2010 through 31 March 2026. FAERS contains voluntary spontaneous reports of suspected drug-related adverse events submitted by healthcare professionals, consumers, and manufacturers. Analyses were performed on aggregated, de-identified public-domain data; institutional review board approval was not required for the database component. Duplicate reports were identified and removed on the basis of case identifier (caseid) and latest report-receipt date, retaining only the most recent version of each individual case safety report in accordance with standard FAERS curation practice. Per openFDA documentation, FAERS report coverage nominally extends to 2004 although earlier quarters contain sparse records; our query (endpoint ‘/drug/event.json’, no explicit receipt-date filter) returned reports with receipt dates from Q1 2010 onwards within the specified window. The observed 2010 lower bound reflects the earliest receipt date returned by our 22-drug exact-match query; records prior to 2010 are present in the openFDA archive but did not match our drug-specific inclusion criteria. Reporting of the present observational analysis was informed by the STROBE principles for transparent reporting of observational research and the READUS-PV recommendations for transparent reporting of disproportionality analyses of individual case safety reports (Fusaroli, Salvo, Khouri, Raschi et al. Drug Safety 2024; 47:585–599); the completed STROBE and READUS-PV checklists are provided as Supplementary Tables S6, S7, respectively.

### 2.2 Drug selection and synonyms

Twenty-two VEGFi/VEGFRi agents were selected *a priori* based on mechanistic coherence and clinical availability, comprising 6 monoclonal antibodies or decoy receptors (bevacizumab, ramucirumab, aflibercept, ranibizumab, brolucizumab, conbercept) and 16 VEGFR tyrosine kinase inhibitors. For each drug, up to six synonyms (international nonproprietary name, brand names, and investigational codes) were queried as uppercase exact matches against three openFDA fields joined by Boolean OR: patient. drug.openfda.generic_name.exact, patient. drug.openfda.brand_name.exact, and patient. drug.medicinalproduct.exact. Only reports with drugcharacterization = 1 (primary suspect) were included in the primary analysis in order to improve specificity for suspected causative attribution; the potential sensitivity trade-off is discussed in the Limitations. The complete synonym dictionary is provided as Supplementary Table S1 (Online Resource). This strategy was designed to mitigate variability in drug naming, but misspelled or uncoded free-text entries may still have been missed.

### 2.3 Outcome ascertainment

PRES events were identified using a MedDRA-based case definition applied at the Preferred Term (PT) level (Supplementary Table S2). The narrow (primary) definition comprised “Posterior reversible encephalopathy syndrome” and “Reversible posterior leukoencephalopathy syndrome”. The broad definition, used only for sensitivity analysis S1, additionally included “Hypertensive encephalopathy”, “Leukoencephalopathy”, and “Toxic leukoencephalopathy”. Because these broad terms are less specific for the clinico-radiological PRES entity, they were confined to sensitivity analyses and not used for primary signal estimation.

### 2.4 Disproportionality analysis

Four complementary disproportionality metrics were computed for each drug from the 2 × 2 contingency table {a = drug-of-interest and PRES event, b = drug-of-interest and non-PRES event, c = comparator and PRES event, d = comparator and non-PRES event}, with N = a + b + c + d. The unit of analysis was the deduplicated individual case safety report (ICSR). For ICSRs listing more than one eligible primary-suspect drug, each eligible primary-suspect drug independently contributed to its own drug-specific 2 × 2 table (i.e., the same ICSR could enter the 2 × 2 tables of multiple drugs as a drug-of-interest report). The denominator N therefore equals the total number of deduplicated primary-suspect ICSRs in FAERS within the query window (19,980,897), and the count ‘c' for any given drug-of-interest is the number of PRES-coded ICSRs where the drug-of-interest was not listed as primary suspect. In the primary analysis, comparators comprised all other primary-suspect FAERS drugs in the database; sensitivity analysis S4 restricted comparators to the VEGFi/VEGFRi class only.

- Reporting Odds Ratio (ROR): ROR = ad/bc, with 95% CI from standard error SE_ln = √(1/a + 1/b + 1/c + 1/d). Signal threshold: lower 95% CI > 1 AND a ≥ 3.
- Proportional Reporting Ratio (PRR): PRR = [a/(a + b)]/[c/(c + d)], with χ^2^ = N (ad − bc)^2^/[(a + b) (c + d) (a + c) (b + d)]. Signal threshold: PRR ≥ 2 AND χ^2^ ≥ 4 AND a ≥3.
- Bayesian Confidence Propagation Neural Network Information Component (BCPNN-IC): IC = log_2_ [aN/((a + b) (a + c))] with Var(IC) = (1/ln2^2^) (1/a − 1/(a + b) − 1/(a + c) + 1/N); IC_025_ = IC − 1.96·√Var(IC) (Bate et al., 1998). Signal threshold: IC_025_ > 0.
- Empirical Bayes Geometric Mean (EBGM): Bate-Evans approximation with α = β = 0.5 gamma prior; EBGM = (a + 0.5)/(E + 0.5), where E = (a + b) (a + c)/N (DuMouchel, 1999). Signal threshold: EBGM ≥ 2.

A drug was classified as having a robust signal if at least three of the four methods met their respective thresholds.

To control for multiple testing across the 16 agents with at least one PRES report, two-sided Fisher exact tests were computed for each drug–PRES 2 × 2 table and corrected by both the Benjamini–Hochberg false-discovery-rate procedure (q < 0.05) and the Bonferroni family-wise procedure (α = 0.05/16; a conservative bound at α = 0.05/88, corresponding to 22 agents × 4 methods, is additionally reported). Fisher exact tests were used in preference to the asymptotic approximation because several agents had small report counts (tivozanib n = 12, vandetanib n = 5, apatinib n = 1). The *ad hoc* ≥3-of-4-method rule was replaced by a principled definition: a robust elevated signal must (i) survive FDR correction at q < 0.05 and (ii) have a positive Bayesian information component lower bound (IC_025_ > 0). Under this definition 13 of the 16 evaluable agents are robust signals (Supplementary Table S11).

### 2.5 Exploratory reporting-interval analysis (weibull)

For PRES-positive primary-suspect reports with both drugstartdate and receivedate populated and internally consistent (drug start ≤ report receipt; interval ≤5 years), the reporting interval in days was computed. Of the 1,128 PRES-positive primary-suspect reports for the 22 agents, 445 (39%) had both dates populated and internally consistent and contributed to this estimate; the remaining 61% were excluded for missing or inconsistent dates. A single Weibull distribution was fit to the pooled reporting intervals of all eligible PRES-positive reports across all contributing agents, using maximum likelihood estimation (survreg function, R survival package), treating all observations as uncensored. Per-drug Weibull parameters were not separately estimated, because for several agents the eligible primary-suspect PRES counts (e.g., tivozanib n = 12, nintedanib n = 5) were too small for stable per-drug shape estimation. The reported shape parameter β and its 95% confidence interval therefore describe the pooled across-drugs reporting-delay distribution and should be interpreted as a class-level descriptor of the timing with which PRES events for VEGFi/VEGFRi are reported to FAERS, not as a per-drug hazard function or time-to-event analysis. β < 1 indicates front-loaded reporting; β = 1 a roughly time-independent reporting pattern; β > 1 increasing reporting delay over time. This interval captures drug start to FDA receipt rather than drug start to true clinical event onset, reflecting the standard FAERS convention when case narratives are not available.

### 2.6 Subgroup and sensitivity analyses

Pre-specified subgroup analyses stratified the primary signal by sex, reporter qualification (physician vs. consumer), and geographic region. Six prespecified sensitivity analyses were conducted: (S1) broad PRES PT definition; (S2) physician-reported cases only; (S3) post-2014 time window; (S4) within-class comparator using only VEGFi/VEGFRi reports as denominator; (S5) leave-one-out at the drug level; (S6) pooled highly-selective vs. multi-kinase TKI comparison.

### 2.7 VEGFR-2 selectivity: categorical contrast and continuous correlation

To evaluate Hypothesis 2 under a continuous rather than categorical model, published VEGFR-2 biochemical IC_50_ values (KDR enzymatic assay; nanomolar) were retrieved for the 11 VEGFR-TKIs for which a computable ROR was obtained. Values were extracted from the original drug-discovery manuscripts (Supplementary Table S4) (Nakamura et al., 2006; Hu-Lowe et al., 2008; Yakes et al., 2011; Matsui et al., 2008; Sun et al., 2014; Wilhelm et al., 2011; Kumar et al., 2007; Mendel et al., 2003; Wilhelm et al., 2004; Hennequin et al., 2002; Hilberg et al., 2008). The per-agent selective-versus-multi-kinase assignment rests on these published VEGFR-2 IC_50_/kinome-selectivity values and their literature sources, tabulated per agent in Supplementary Table S4. As a primary, exclusion-free test of the selectivity hypothesis, VEGFR-TKIs were classified *a priori* as highly selective (tivozanib, lenvatinib, fruquintinib, axitinib) or multi-kinase (cabozantinib, regorafenib, pazopanib, vandetanib, sunitinib, sorafenib) on the basis of their published kinome profiles, with nintedanib retained as a separate FGFR-primary agent; ROR was compared between subclasses using the one-sided Wilcoxon–Mann–Whitney rank-sum test, and the association between binary selectivity status and ROR across all 11 VEGFR-TKIs was quantified by Spearman correlation. As a secondary continuous model, Spearman’s rank correlation coefficient was computed between log_10_ IC_50_ and log_10_ ROR. To assess robustness given the small number of agents (n = 11), two complementary robustness analyses were prespecified: (i) non-parametric bootstrap 95% confidence intervals for ρ using 2,000 resamples with replacement; and (ii) leave-one-out at the drug level, in which each of the 11 drugs was iteratively removed and ρ recomputed on the remaining 10. Two further sensitivity exclusions were prespecified on pharmacological grounds prior to analysis: (i) cabozantinib, because its primary clinical target as listed on the United States Food and Drug Administration product label is MET rather than VEGFR-2; and (ii) nintedanib, because its kinome profile is FGFR-primary and its licensed indication (idiopathic pulmonary fibrosis) lies outside the oncology use-case that generates the majority of comparator exposure. Monoclonal antibodies and decoy receptors were excluded from this analysis because they target the VEGF ligand, not the VEGFR-2 kinase, and no directly comparable biochemical IC_50_ exists. Robustness analysis results are presented in Supplementary Table S8 (Online Resource).

We assigned agents *a priori* to the selective versus multikinase VEGFR-TKI groups defined above on the basis of published kinase-selectivity and target-affinity profiles, not by any *post hoc* or data-driven procedure: selective agents were those with predominant VEGFR-1/2/3 inhibition and limited off-target kinase activity, whereas multikinase agents engaged substantial additional kinase targets. Anti-VEGF biologics and intravitreal agents were analysed separately. Because lenvatinib (VEGFR-1/2/3 plus FGFR1–4, PDGFRα, RET, and KIT) lies at the selective/multikinase boundary, its group assignment was examined in a sensitivity analysis (Supplementary Table S9).

### 2.8 Literature concordance scan

To cross-validate the FAERS signal ranking against the peer-reviewed case-report literature, a targeted PubMed search was executed on 20 April 2026 for each of the 13 VEGFi/VEGFRi agents with a computable ROR. The search syntax was <drug_INN> AND (“posterior reversible encephalopathy syndrome” OR “PRES” OR “reversible posterior leukoencephalopathy” OR “RPLS”) executed directly against the PubMed database (https://pubmed.ncbi.nlm.nih.gov). English-language PubMed-indexed original case reports, case series, and case-inclusive reviews published 2004–2026 were counted per drug (Supplementary Table S3). Conference abstracts not indexed by PubMed and non-English literature were recorded but not included in the primary count. Spearman’s rank correlation was computed between FAERS ROR and published case-report count. The four PubMed-indexed fruquintinib-associated PRES cases identified by this search are summarised as focused literature context (Table 1); this is a narrative literature summary, not a systematic review or a consecutive case series.

**TABLE 1:** Focused literature context: published PubMed-indexed cases of fruquintinib-associated posterior reversible encephalopathy syndrome (narrative literature summary, not a systematic review).

Feature	Wang et al. (2023)	Ledet et al. (2025)	ElManfalouty et al. (2025)	Ioannidis et al. (2025)
Age/sex	44/F	41/F	71/F	68/M
Primary cancer	mCRC (colon)	mCRC (rectal)	mCRC (colon)	mCRC (sigmoid)
Fruquintinib duration before onset	∼4 weeks	∼3 weeks (day 21)	Week 4	4 days
Peak BP (mmHg)	Hypertensive*	Hypertensive*	140/90 (normotensive)	Normotensive on admission
Presenting symptoms	Visual disturbance, headache, seizure	Headache, blurred vision, hypertensive crisis	Cortical visual impairment (normotensive)	Coma (GCS 5/15), headache, vomiting, generalized tonic-clonic seizure
MRI pattern	Bilateral parieto-occipital T2/FLAIR hyperintensity	Bilateral parieto-occipital cortical-subcortical T2/FLAIR hyperintensity	Bilateral occipital cortical-subcortical T2/FLAIR hyperintensity	Bilateral parieto-occipital cortical–subcortical T2/FLAIR hyperintensity; DWI negative; no gadolinium enhancement
Management	Drug withdrawal; antihypertensives	Drug withdrawal; antihypertensives	Drug withdrawal; antihypertensives; corticosteroids	Drug withdrawal; levetiracetam; aspirin
Time to clinical recovery	∼2 weeks	∼3–4 weeks	∼2 weeks	Consciousness within hours; discharged symptom-free
MRI follow-up resolution	Complete	Complete	Not reported	Complete at 4 weeks
Rechallenge	Not attempted	Not attempted	Not attempted	Full-dose rechallenge under BP < 130/80 mmHg; no recurrence at 3 months

BP, blood pressure; CRC, colorectal cancer; FLAIR, fluid-attenuated inversion recovery; HTN, hypertension; mCRC, metastatic colorectal cancer; MRI, magnetic resonance imaging; PRES, posterior reversible encephalopathy syndrome; VEGFR, vascular endothelial growth factor receptor.

* Hypertensive crisis/severe elevation reported qualitatively in the source publication; exact mmHg values not specified in the published abstract.

### 2.9 Statistical software and reproducibility

All analyses were performed in R version 4. Disproportionality metrics were implemented from 2 × 2 tables to enable transparent calculation. Weibull fitting used the survival package. Spearman correlations were computed with the cor.test function. Figures were generated with ggplot2. All code and aggregated data supporting this analysis are available from the corresponding author on reasonable request and will be deposited at a persistent DOI upon acceptance.

## 3 Results

### 3.1 Overall report volumes

Between 1 January 2010 and 31 March 2026, 19,980,897 primary-suspect reports were identified in FAERS, of which 9,745 contained a PRES-related MedDRA PT (narrow definition). Across the 22 VEGFi/VEGFRi agents analysed, 420,386 reports listed a VEGFi/VEGFRi as primary suspect, with 1,128 of these reports coded for PRES. Bevacizumab contributed the largest single-drug share of PRES reports (n = 532, 47% of VEGFi-class PRES), followed by lenvatinib (n = 170) and axitinib (n = 89). Six agents—anlotinib, brolucizumab, conbercept, brivanib, cediranib, and surufatinib—recorded zero primary-suspect PRES reports in the query window (Table 2); this reflects FAERS post-marketing geography (several are China-primary or developmentally discontinued agents) rather than established absence of risk, and is discussed further in the Limitations.

**TABLE 2:** Characteristics of the 22 VEGFi/VEGFRi agents analysed and their primary-suspect FAERS report volumes (2010-Q1 to 2026-Q1).

Drug (INN)	Mechanistic class	Primary-suspect reports (n)	PRES reports (n)
Bevacizumab	mAb/decoy	123,868	532
Cabozantinib	TKI multi-kinase	43,248	42
Sunitinib	TKI multi-kinase	41,426	70
Aflibercept	mAb/decoy	31,131	30
Lenvatinib	TKI selective	30,651	170
Nintedanib	TKI (IPF-primary; also oncology)	30,088	5
Pazopanib	TKI multi-kinase	26,692	78
Ranibizumab	Intravitreal	26,172	1
Sorafenib	TKI multi-kinase	20,487	27
Axitinib	TKI selective	18,965	89
Regorafenib	TKI multi-kinase	11,351	27
Ramucirumab	mAb/decoy	6,001	22
Brolucizumab	Intravitreal	3,561	0
Fruquintinib	TKI selective	3,083	17
Vandetanib	TKI multi-kinase	1,750	5
Tivozanib	TKI selective	1,488	12
Apatinib	TKI (China)	318	1
Cediranib	TKI (invest.)	73	0
Surufatinib	TKI (China)	21	0
Conbercept	Intravitreal	6	0
Brivanib	TKI (invest.)	6	0
Anlotinib	TKI (China)	0	0

INN, international nonproprietary name; IPF, idiopathic pulmonary fibrosis; PRES, posterior reversible encephalopathy syndrome; TKI, tyrosine kinase inhibitor.

### 3.2 Primary disproportionality signals

**FIGURE 1:**
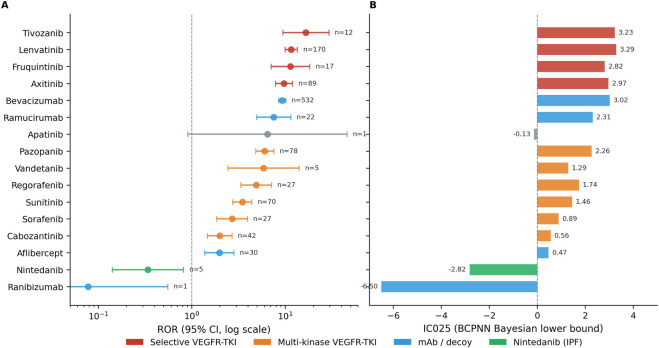
Multi-method disproportionality for PRES across the 16 VEGFi/VEGFRi agents with at least one primary-suspect PRES report (a ≥ 1). **(A)** Reporting Odds Ratio (ROR) with 95% confidence intervals on a log scale; drugs ordered by ROR. **(B)** Information Component lower 95% credibility bound (IC_025_) from the BCPNN method. Colours encode mechanistic class as indicated in the legend. The dashed vertical lines indicate signal thresholds (ROR = 1; IC_025_ = 0). Drugs with zero primary-suspect PRES reports (anlotinib, brolucizumab, conbercept, brivanib, cediranib, surufatinib) are omitted from this figure; all 22 agents, including these six, are listed in Table 2.

Four disproportionality methods were computed for each drug (Table 3; Figure 1). Of the 16 evaluable agents, 13 met the prespecified primary criterion for a robust elevated signal (Benjamini–Hochberg FDR q < 0.05 and a positive Bayesian IC_025_; §2.4, Supplementary Table S11), with elevated ROR up to 16.68; nintedanib produced a separate, significantly inverse signal (ROR 0.34). The earlier ≥3-of-4 screening rule, which 11 agents met, is retained only as a labelled sensitivity check. The strongest signal was generated by tivozanib (ROR 16.68, 95% CI 9.45–29.45; IC_025_ = 3.23; n = 12), followed by lenvatinib (ROR 11.62, 9.98–13.52; n = 170), fruquintinib (ROR 11.38, 7.06–18.34; n = 17), axitinib (ROR 9.74, 7.90–12.01; n = 89), and bevacizumab (ROR 9.29, 8.51–10.14; n = 532). Nintedanib produced a significantly inverse signal (ROR 0.34, 0.14–0.82; IC_025_ = −2.82; n = 5).

**TABLE 3:** Four-method disproportionality analysis of PRES for the 16 VEGFi/VEGFRi agents with at least one primary-suspect PRES report (a ≥ 1). The remaining six agents (anlotinib, brolucizumab, conbercept, brivanib, cediranib, surufatinib) had zero primary-suspect PRES reports in the query window and are listed in Table 2 with their total primary-suspect exposure volumes.

Drug	n	ROR (95% CI)	PRR	IC_025_	EBGM	Robust signal
Tivozanib	12	16.68 (9.45–29.45)	16.55	3.23	10.20	Positive
Lenvatinib	170	11.62 (9.98–13.52)	11.56	3.29	11.04	Positive
Fruquintinib	17	11.38 (7.06–18.34)	11.32	2.82	8.73	Positive
Axitinib	89	9.74 (7.90–12.01)	9.70	2.97	9.18	Positive
Bevacizumab	532	9.29 (8.51–10.14)	9.26	3.02	8.74	Positive
Ramucirumab	22	7.56 (4.97–11.49)	7.53	2.31	6.57	Positive
Apatinib	1	6.47 (0.91–46.05)	6.45	−0.13	2.29	Not robust
Pazopanib	78	6.05 (4.84–7.56)	6.03	2.26	5.81	Positive
Vandetanib	5	5.87 (2.44–14.14)	5.86	1.29	4.06	Positive
Regorafenib	27	4.90 (3.36–7.15)	4.89	1.74	4.56	Positive
Sunitinib	70	3.49 (2.76–4.41)	3.48	1.46	3.41	Positive
Sorafenib	27	2.71 (1.86–3.95)	2.71	0.89	2.62	Positive
Cabozantinib	42	2.00 (1.47–2.70)	2.00	0.56	1.97	Positive
Aflibercept	30	1.98 (1.38–2.83)	1.98	0.47	1.94	Positive
Nintedanib	5	0.34 (0.14–0.82)	0.34	−2.82	0.36	Inverse
Ranibizumab	1	0.08 (0.01–0.56)	0.08	−6.50	0.11	Not robust

Abbreviations: CI, confidence interval; EBGM, empirical bayes geometric mean; IC_025_, lower 95% credibility interval of the Information Component; n, PRES reports with the drug as primary suspect; PRR, proportional reporting ratio; ROR, Reporting odds ratio. Drugs with no primary-suspect PRES reports (brolucizumab, conbercept, anlotinib, cediranib, surufatinib, brivanib) are omitted.

Every agent that met the earlier ≥3-of-4 screening also survived both Benjamini–Hochberg FDR (q < 0.05) and Bonferroni correction; the primary definition (FDR-significant with IC_025_ > 0) classifies 13 of the 16 evaluable agents as robust, adding cabozantinib and aflibercept, the two lowest-ROR robust signals. The four selective VEGFR-2 TKIs and bevacizumab survived even the most conservative Bonferroni bound (α = 0.05/88) by many orders of magnitude (Fisher exact p from 2 × 10^−11^ to < 10^–300^). Apatinib (n = 1) survived no correction. The nintedanib inverse signal remained FDR-significant (q = 0.009) but not family-wise significant. Per-agent results are in Supplementary Table S11.

### 3.3 Test of H1 (class-wide signal)

The robust positive signals encompass all systemically administered oncology VEGFi/VEGFRi agents in our drug set (bevacizumab, lenvatinib, axitinib, pazopanib, sunitinib, sorafenib, regorafenib, ramucirumab, fruquintinib, tivozanib, vandetanib, cabozantinib); aflibercept also met the multiplicity-corrected criterion, although its FAERS reports combine the systemic (ziv-aflibercept) and intravitreal formulations. Notably, anlotinib and surufatinib contributed zero FAERS PRES reports over the query window, and apatinib contributed only a single report (n = 1, below the a ≥ 3 threshold for robust signal detection); this observation is discussed in the Limitations in light of published Chinese case-report evidence for anlotinib (Nan et al., 2021) and is attributed primarily to the post-marketing geography of these agents. The null or inverse signals among the intravitreal anti-VEGF agents (Section 3.6) contrast with these positive systemic signals and are interpreted as supporting the class-level VEGF-pathway–PRES hypothesis while conditioning it on systemic exposure (H4).

### 3.4 Selectivity-gradient analysis

Disproportionality signal intensity separated cleanly between the highly selective and multi-kinase VEGFR-TKI subclasses. Every highly selective VEGFR-TKI (tivozanib ROR 16.68, lenvatinib 11.62, fruquintinib 11.38, axitinib 9.74) ranked above every multi-kinase VEGFR-TKI (pazopanib 6.05, vandetanib 5.87, regorafenib 4.90, sunitinib 3.49, sorafenib 2.71, cabozantinib 2.00) — a separation that was robust across classification choices (median ROR 11.50 vs. 4.19). This categorical contrast was statistically significant by a rank-based test (Mann–Whitney U, selective vs. multi-kinase, one-sided p = 0.005; selective vs. all other VEGFR-TKIs including nintedanib, p = 0.003), and selectivity status correlated with ROR across all 11 VEGFR-TKIs (Spearman ρ = 0.84, p = 0.001, no drugs excluded). This categorical separation was reproduced across all four disproportionality metrics (ROR, PRR, IC_025_, and EBGM; Mann–Whitney p = 0.005 in each) and in every drug-level leave-one-out iteration (p ≤ 0.012 throughout); it was sensitive only to reclassification of lenvatinib—a VEGFR-selective agent with appreciable FGFR activity—into the multi-kinase group, which preserved the contrast for ROR (p = 0.033) but reduced it to borderline for IC_025_ (p = 0.058), a sensitivity we report explicitly (§4.8). Because comparable, assay-harmonised IC_50_ values were available for only a subset of agents and are sensitive to assay conditions, the continuous IC_50_–ROR relationship (Supplementary Material, Supplementary Figure S2 and Supplementary Table S8) is presented as exploratory and hypothesis-generating only and does not inform our clinical interpretation, which rests on the categorical selectivity classification. The robust categorical separation, alongside the fragile continuous correlation, indicates that the selectivity signal resides in the categorical selective-versus-multi-kinase contrast rather than in a graded relationship with biochemical potency—a distinction consistent with the null continuous receptor-affinity association reported by Donati et al. (2026).

In a sensitivity analysis varying the classification of lenvatinib (Supplementary Table S9), selective VEGFR-TKIs showed significantly higher PRES reporting than multikinase inhibitors under every classification choice (Mann–Whitney one-sided p = 0.005–0.033). Complete rank separation held when lenvatinib was classified as selective (primary analysis) or excluded, but was lost when lenvatinib was reclassified as multikinase, because its own reporting signal (ROR ≈11.4) then became the highest value within the multikinase group.

### 3.5 Exploratory reporting-interval distribution

A single Weibull distribution fit to the pooled reporting intervals across all PRES-positive primary-suspect reports (n = 445 eligible reports across all contributing agents; Section 2.5) returned a shape parameter β = 0.83 (asymptotic 95% confidence interval 0.78–0.89, derived from the maximum-likelihood standard error of the pooled model) and scale parameter η = 185 days, consistent with a front-loaded class-level reporting-interval distribution. The narrow asymptotic confidence interval reflects the precision of the pooled estimate and should not be interpreted as drug-specific robustness.

### 3.6 Test of H4 (route and indication effects)

Intravitreal anti-VEGF agents returned no positive disproportionality signal meeting our pre-specified threshold (a ≥ 3). Ranibizumab had only 1 PRES case across 26,172 primary-suspect reports (point-estimate ROR ≈ 0.08 from the 2 × 2 table, reported for completeness only; below threshold for inferential interpretation), and brolucizumab and conbercept had zero PRES cases. This pattern is consistent with negligible systemic VEGFR-2 blockade at intravitreal doses and provides an internal negative control for the systemic-exposure component of the mechanism (H4a). For indication (H4b), nintedanib—the only predominantly IPF-indicated VEGFR inhibitor in the cohort—produced a significantly inverse signal (ROR 0.34, 95% CI 0.14–0.82; n = 5). The direction of this signal is interpreted in the Discussion (§4) as more likely reflecting indication-channelling and reporter behaviour than a genuine inverse biological association.

### 3.7 Subgroup and sensitivity analyses

Sex-stratified analyses confirmed the class signal in both males and females, with the selectivity gradient preserved in both strata. Physician-reported cases only (S2) produced concordant ROR estimates to the primary analysis, with all eleven prespecified screening-positive signals remaining positive. Restricting the time window to post-2014 (S3) did not materially change drug rankings. Within-class comparator analysis (S4) using only VEGFi/VEGFRi reports as the denominator preserved the selectivity-gradient ranking. Broad PT definition (S1) increased the count of PRES reports by approximately 20% without substantially altering ROR ranks. Summary results of the four tabulated sensitivity analyses (S1–S4) are provided in Supplementary Table S5; the leave-one-out (S5) and pooled selective-versus-multi-kinase comparison (S6) are reported in §3.4.

### 3.8 Literature concordance scan

A targeted PubMed scan identified published PRES case reports across the 13 VEGFi/VEGFRi with a computable ROR (Supplementary Table S3). Spearman’s rank correlation between FAERS ROR and published case-report volume was effectively null (ρ = 0.09, p = 0.78). Two high-ranked FAERS signals were essentially absent from the PubMed-indexed literature: tivozanib (0 PubMed case reports; one non-indexed conference abstract at SHM Converge 2022) and ramucirumab (no dedicated PubMed-indexed case report identified). Bevacizumab dominated the published literature with ≥20 PubMed-indexed case reports (2006–present), despite ranking fifth in FAERS ROR.

### 3.9 Focused literature context: published fruquintinib-associated PRES cases

To provide focused clinical context for the fruquintinib disproportionality signal, we summarise the four PubMed-indexed cases of fruquintinib-associated PRES identified by our structured search (Wang et al., 2023; Ledet et al., 2025; ElManfalouty et al., 2025; Ioannidis et al., 2025) (Table 1). Across these four patients (ages 41–71; three of four female), onset ranged from treatment day 4 to approximately week 4 of fruquintinib, broadly consistent with the front-loaded class-level reporting-interval distribution observed in the present FAERS analysis (§3.5); the shortest interval (treatment day 4 in Ioannidis et al. (2025), presenting with coma and generalized tonic-clonic seizure) illustrates that clinically severe PRES can emerge within the first week of exposure. Severe new-onset hypertension preceded neurological symptoms in some but not all cases—the Cureus report (ElManfalouty et al., 2025) and the Ioannidis 2025 case (Ioannidis et al., 2025) were normotensive—indicating that a meaningful subset develops without a hypertensive intermediary. Bilateral parieto-occipital cortical–subcortical T2/FLAIR hyperintensity was the dominant neuroimaging pattern. Management was uniform in principle (immediate fruquintinib withdrawal with blood-pressure control), and clinical recovery was complete in all four cases. Rechallenge was attempted in two with divergent outcomes: reduced-dose rechallenge (Wang et al., 2023) produced recurrence within 1 week, whereas full-dose rechallenge under strict blood-pressure control (Ioannidis et al., 2025) was tolerated without recurrent serious events at 3 months. This focused literature summary is descriptive context for the disproportionality signal and is not a systematic review or a consecutive case series.

## 4 Discussion

### 4.1 Principal findings

In this within-class disproportionality analysis of 22 VEGFi/VEGFRi in FAERS, we identified robust elevated PRES reporting signals for 13 of the 16 evaluable agents (elevated ROR up to 16.68), together with one significantly inverse signal (nintedanib, ROR 0.34). Four findings appear most informative. First, signal intensity separated categorically by VEGFR-2 selectivity: every highly selective VEGFR-TKI ranked above every multi-kinase agent (Mann–Whitney p = 0.005; selectivity–ROR ρ = 0.84, p = 0.001), and this separation was reproduced across all four disproportionality metrics (ROR, PRR, IC_025_, EBGM) and every leave-one-out iteration; the continuous IC_50_ correlation, by contrast, was not robust and is reported as hypothesis-generating only. Second, route of administration eliminated the disproportionality signal entirely for intravitreal anti-VEGF agents, providing an internal negative control that is consistent with the systemic-exposure component of the mechanism. Third, nintedanib—a multi-kinase VEGFR inhibitor—returned a significantly inverse signal (ROR 0.34) whose interpretation requires careful separation of biology from indication. Fourth, two of the top six disproportionality signals—tivozanib (ROR 16.68) and ramucirumab (ROR 7.56) — were essentially absent from the dedicated PubMed-indexed case-report literature, identifying them as high-priority targets for confirmatory replication.

### 4.2 Interpreting the selectivity-gradient signal

**FIGURE 2:**
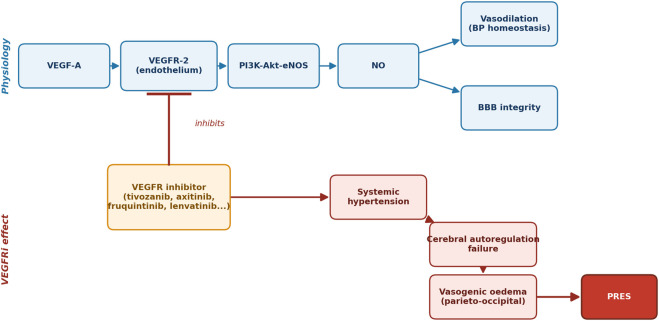
Mechanistic framework for VEGFR inhibitor-associated PRES. Upper row (blue): physiological VEGF-A/VEGFR-2/PI3K-Akt-eNOS/NO axis and its downstream effects on vasodilation, blood pressure homeostasis, and blood-brain barrier integrity. Lower row (red): pathological cascade activated when a VEGFR inhibitor (orange box) blocks VEGFR-2 signalling—reduced NO → systemic hypertension → cerebral autoregulation failure → parieto-occipital vasogenic oedema → PRES.

VEGF-A acting through VEGFR-2 on the vascular endothelium helps maintain endothelial homeostasis via PI3K–Akt–eNOS-mediated nitric oxide production, preserving both arterial tone and blood–brain barrier integrity (Ferrara et al., 2003). Pharmacological VEGFR-2 blockade can reduce endothelial nitric oxide availability, provoke systemic hypertension, and contribute to posterior vasogenic oedema, providing a biologically plausible backdrop for PRES (Figure 2) (Hinchey et al., 1996; Eremina et al., 2008). The disproportionality ranking observed here is compatible with this framework: the four highly selective VEGFR-TKIs (tivozanib, lenvatinib, fruquintinib, axitinib) generated the strongest signals and, as a group, ranked above the multi-kinase agents that additionally engage PDGFR, RET, and c-KIT. This categorical selective-versus-multi-kinase contrast was robust and required no *post hoc* exclusion (Mann–Whitney p = 0.005; selectivity–ROR ρ = 0.84, p = 0.001 across all 11 VEGFR-TKIs). The continuous test of the same gradient, however, was not robust: the Spearman correlation between log VEGFR-2 IC_50_ and log-ROR was non-significant in the primary analysis (ρ = −0.38, p = 0.25), and the significant correlations obtained after prespecified exclusions (ρ = −0.77 after excluding cabozantinib; ρ = −0.88 after additionally excluding nintedanib) were methodologically fragile, with leave-one-out analysis (Supplementary Table S8) preserving p < 0.05 in only the cabozantinib-removed iteration. This dissociation—a robust categorical contrast alongside a fragile continuous correlation—is itself informative and aligns with the null continuous receptor-affinity association reported by Donati et al. (2026): the selectivity signal appears to reside in the qualitative selective-versus-multi-kinase distinction rather than in a graded relationship with biochemical potency. Moreover, the IC_50_ values are aggregated from heterogeneous published assay systems with different KDR constructs, ATP concentrations, and protocols, and are compared with spontaneous-reporting patterns rather than patient-level exposure data. We therefore interpret the categorical separation as the more dependable observation and the continuous IC_50_–ROR relationship as exploratory and hypothesis-generating. Cohort studies with measured systemic exposure and longitudinal blood-pressure data would be required to test a graded mechanistic dose-response directly.

### 4.3 Route of administration and the intravitreal null

A strong internal control for the VEGF–PRES hypothesis is provided by the three intravitreal anti-VEGF agents—ranibizumab, brolucizumab, and conbercept—for which no positive disproportionality signal was detected despite extensive global post-marketing exposure. Ranibizumab recorded only one PRES case across 26,172 primary-suspect reports (below our a ≥3 threshold); brolucizumab and conbercept recorded none. Intravitreal dosing produces negligible systemic plasma concentrations and, consequently, no meaningful VEGFR-2 blockade outside the eye. This pattern supports, but does not by itself confirm, the proposition that systemic VEGF-pathway inhibition—rather than any off-target or immunogenic property of the biological molecule itself—is a proximate driver of the signals observed for systemically administered agents. Nevertheless, differences in clinical specialty and reporting behavior across ophthalmology and oncology practice may also contribute to the apparent contrast and should be considered when interpreting this negative-control pattern: ophthalmologists may be less likely to recognise PRES, a neurological syndrome, in patients receiving intravitreal injections; such events may be attributed to hypertension or renal disease rather than to the drug; and the populations differ substantially (older patients with age-related macular degeneration versus younger oncology patients). We therefore present the intravitreal null as supportive evidence rather than as confirmation of a systemic-exposure mechanism.

### 4.4 The inverse signal for nintedanib

Nintedanib was the only systemic VEGFR inhibitor in our analysis to return a significantly inverse disproportionality signal (ROR 0.34). Several non-exclusive mechanisms merit consideration before this finding is interpreted biologically meaningful. First, indication channelling: nintedanib is predominantly used in idiopathic pulmonary fibrosis and systemic-sclerosis-associated interstitial lung disease, populations that differ substantially from the oncology cohorts exposed to the remaining 21 agents—older, rarely co-exposed to cytotoxic chemotherapy or high-dose corticosteroids, and with a different baseline profile of hypertension and organ dysfunction. Second, reporter behaviour: pulmonologists and rheumatologists may be less likely than oncologists to recognize or report PRES as a discrete neurological event, lowering the observed count without changing the underlying biology. Third, pharmacology: nintedanib exhibits markedly weaker VEGFR-2 affinity (IC_50_ ≈ 21 nM) relative to its FGFR and PDGFR activity (Hilberg et al., 2008), and is associated in registrational and real-world data with a substantially lower incidence of clinically significant hypertension than the selective VEGFR-TKIs. We therefore interpret the inverse nintedanib signal as the combined expression of these confounders rather than as evidence of a true inverse biological effect. The point cannot, however, be settled within spontaneous-report data alone; matched-cohort comparisons between IPF and oncology populations would be required to disentangle biology from context.

Every nintedanib PRES report in FAERS carried a non-oncological fibrotic indication (idiopathic pulmonary fibrosis, interstitial lung disease, or systemic sclerosis) and none arose in an oncology population, whereas the selective VEGFR-TKI reports were uniformly oncological (Supplementary Table S10); although the small nintedanib count (n = 5) and the absence of indication-level denominators preclude any formal matched comparison, this is consistent with—but not proof of—both lower vascular selectivity and a different reporting population, and should be tested prospectively. Importantly, the apparent absence of PRES in oncology-exposed nintedanib patients cannot be confirmed from FAERS, because nintedanib is only rarely used in oncology settings where PRES might be more readily recognised and reported.

### 4.5 Reporting-interval distribution

Weibull modeling returned β = 0.83 (95% CI 0.78–0.89) with scale η = 185 days, indicating a front-loaded reporting-delay distribution rather than a true clinical hazard pattern. These descriptive findings do not define a biological safe window, but they support clinical vigilance from the first treatment weeks rather than relaxation of monitoring after an arbitrarily early period.

### 4.6 FAERS signal versus published-literature volume

A targeted PubMed scan was performed to cross-validate our signal ranking against the independent evidence base of peer-reviewed case reports (Supplementary Table S3) (Wang et al., 2023; Ledet et al., 2025; ElManfalouty et al., 2025; Ioannidis et al., 2025; Chelis et al., 2012; Deguchi et al., 2018; Myint et al., 2014; Nakamura et al., 2017; Tseng et al., 2022; Chae et al., 2018). The correlation between FAERS ROR and published case-report volume across the 13 VEGFi/VEGFRi with computable ROR was effectively null (ρ = 0.09, p = 0.78; Supplementary Figure S1). Far from being an unfavourable result, this observation establishes an important epistemic point: pharmacovigilance disproportionality and case-report volume capture different dimensions of drug–event association and are therefore informationally complementary rather than redundant. The decoupling is visible at the two extremes of the ranking. Bevacizumab, which occupies position 5 in our FAERS analysis, dominates the published literature (≥20 PubMed-indexed case reports, 2006–present), a divergence plausibly attributable to its longer on-market duration, early novelty reporting, and Weber-type attenuation of its spontaneous-report rate over time. At the opposite extreme, the top-ranked FAERS signal (tivozanib) and the sixth-ranked signal (ramucirumab) are essentially absent from the PubMed record: for tivozanib we identified zero peer-reviewed PubMed-indexed case reports and a single non-indexed conference abstract (SHM Converge 2022); for ramucirumab we identified no dedicated PubMed-indexed case report. For fruquintinib, by contrast, four published PubMed-indexed case reports have now accumulated (Wang et al., 2023; Ledet et al., 2025; ElManfalouty et al., 2025; Ioannidis et al., 2025), which together represent an emerging but still small case-report literature for this agent. Both signals are therefore under-represented in the peer-reviewed case-report literature despite labelling-level acknowledgement of PRES/RPLS on the US FDA (Fotivda; Cyramza) and EU EMA (Fotivda; Cyramza) product information, supporting prioritisation for confirmatory case-series description; they meaningfully extend the spectrum of VEGFR-associated PRES previously characterised at the drug level (Balcerac et al., 2023; Lei et al., 2024) and for fruquintinib in particular (Xu et al., 2025).

The apparent under-representation of certain agents warrants careful interpretation, because a high disproportionality estimate can coexist with few absolute reports. For tivozanib—the strongest signal (ROR 16.68) yet without any published PRES case report—three non-exclusive explanations apply. First, true event scarcity: tivozanib was approved only in 2021 and contributes a small post-marketing population (1,488 reports), so few absolute PRES events are expected even at a high relative reporting rate. Second, publication and notoriety bias: the absence of indexed tivozanib-PRES cases plausibly lowers clinician awareness and, in turn, spontaneous reporting—a self-reinforcing loop—whereas the extensive bevacizumab-PRES literature elevates recognition and reporting for that agent. Third, reporting-behaviour differences: ramucirumab is given intravenously in oncology clinics, frequently within combination regimens, so PRES may be attributed across co-administered drugs or under-attributed to ramucirumab specifically, diluting its single-agent signal. These mechanisms are not mutually exclusive and cannot be disentangled within spontaneous-reporting data; they are best resolved by targeted active surveillance.

### 4.7 Relation to prior literature

Our findings extend, rather than replicate, the global drug-level PRES signal rankings from VigiBase (Balcerac et al., 2023) and earlier FAERS-wide analyses (Lei et al., 2024), neither of which resolved the VEGFR subclass at the within-class level. The recent single-drug FAERS safety profile for fruquintinib (Xu et al., 2025) confirmed the existence of a neurological signal but did not contextualise it against comparator VEGFR inhibitors. The most directly comparable study is that of Donati et al. (Donati et al., 2026), who analysed 15 anti-VEGF/VEGFR agents for PRES in FAERS and concluded that PRES is probably a class effect. Our results, derived from an overlapping but independently curated dataset, are consistent with that class-effect conclusion and reinforce it. They also complement it in three respects. First, whereas their continuous receptor-affinity regression did not detect an association with VEGFR (only an inverse FGFR4 signal), we show that PRES disproportionality separates robustly along a categorical VEGFR-2 selectivity axis—every highly selective VEGFR-TKI exceeded every multi-kinase agent (Mann–Whitney p = 0.005; ρ = 0.84, p = 0.001 across all 11 VEGFR-TKIs, with no drug excluded) — even though, in agreement with Donati et al., our continuous IC_50_ correlation was not robust. A categorical contrast can hold where a continuous one fails, because the grouping captures the presence or absence of substantial off-target multikinase activity (FGFR, PDGFR, KIT) rather than the precise magnitude of VEGFR-2 affinity; consistent with this, lenvatinib’s appreciable FGFR activity and the inverse FGFR4 signal reported by Donati et al. illustrate why continuous VEGFR-2 affinity alone may not order these agents. Second, we characterise the reporting-interval distribution by Weibull modelling, an analysis that Donati et al. reported they could not perform owing to missing event-date data, providing a complementary temporal description (acknowledging the reporting-interval caveats in §4.5 and §4.8). Third, we benchmark the FAERS ranking against the peer-reviewed case-report literature, identifying tivozanib and ramucirumab as disproportionality signals that are essentially unreported as dedicated cases. By constraining the comparator set to a pharmacologically coherent class and applying harmonised MedDRA-PT-based ascertainment, our analysis isolates inter-agent heterogeneity that is otherwise masked when VEGFi/VEGFRi agents are pooled with unrelated drugs. The within-class approach we adopt here also has a methodological antecedent in the 22-drug hypertension analysis of Kuang et al. (2024), extending that template to a distinct neurological endpoint. The two FAERS PRES analyses are therefore complementary rather than redundant.

### 4.8 Limitations

This study shares the structural limitations of all spontaneous-report disproportionality analyses. Spontaneous reporting databases such as FAERS cannot establish causality between exposure and adverse event, nor estimate the true incidence of PRES in any given drug-exposed population, and the disproportionality measures reported here should be interpreted as reporting signal intensity rather than as risk estimates. Reporting rates are subject to notoriety and Weber effects, indication channelling, and differential under-reporting across specialties, countries, and reporting years; the denominators required to estimate true incidence are unavailable within FAERS. MedDRA coding for PRES is imperfect—related clinical narratives may be captured under hypertensive encephalopathy, leukoencephalopathy, or cortical blindness—and we cannot exclude residual misclassification despite our use of harmonised narrow and broad definitions. Confounding by indication and by co-administered agents (cytotoxic chemotherapy, corticosteroids, calcineurin inhibitors), as well as by hypertension and renal dysfunction, cannot be removed within a spontaneous-report framework. Because the primary comparator was the entire FAERS database, the reporting odds ratios reflect relative reporting against a background dominated by unrelated indications and reporting practices, so indication channelling, differential reporting by specialty, and notoriety effects can all influence the estimates; the within-class contrasts reported here (selective versus multi-kinase, and intravitreal versus systemic) are less vulnerable to this than the whole-database odds ratio, because they compare agents reported within broadly similar oncology reporting contexts. Several signals also rest on small report counts (for example, tivozanib n = 12, vandetanib n = 5, apatinib n = 1), for which the confidence intervals are correspondingly wide (tivozanib ROR 16.68, 95% CI 9.45–29.45); point estimates from such sparse data should be read as signal indicators rather than precise effect sizes, which is why Fisher exact tests were used for these agents and apatinib (n = 1) is classified as not robust under any correction, and the within-class rank separation by selectivity does not depend on these sparse high-ROR point estimates because it is driven by the ordering across agents and confirmed by a distribution-free test. A specific limitation for the present class is the geographic underrepresentation of VEGFR inhibitors whose primary market is China: anlotinib contributed zero FAERS reports in our analysis despite a published PRES case in the Chinese oncology literature (Nan et al., 2021), indicating that our null findings for anlotinib, conbercept, and surufatinib, and the low-count non-robust finding for apatinib (n = 1), reflect FAERS coverage as much as drug biology. The reporting-interval analysis captures drug-start to FDA receipt date rather than drug-start to true event onset and should therefore be interpreted as a reporting-delay descriptor, not as a clinical hazard function or a time-to-event analysis; the asymptotic 95% confidence interval reported for the Weibull shape parameter reflects the precision of a single pooled across-drugs model, not drug-specific robustness. Because fewer than half of the eligible reports (445 of 1,128; 39%) carried usable drug-start and receipt dates, the reporting-interval distribution is additionally subject to a date-completeness selection bias and to possible inaccuracy of the recorded date fields, which may vary by reporter type and over time, and the narrow pooled confidence interval should not be interpreted as evidence that individual drugs share the same reporting-delay distribution. Several further limitations of the present analysis warrant explicit acknowledgement. First, the disproportionality analysis was restricted to primary-suspect reports only; an “any-suspect” sensitivity analysis was not performed in the current study but represents a natural extension when external replication is undertaken. Because primary-suspect restriction is conservative for combination-administered agents such as ramucirumab, an any-suspect re-analysis would be expected to strengthen rather than weaken these positive signals. Second, the categorical selectivity contrast, although robust across all four disproportionality metrics and every leave-one-out iteration, rests on a small number of agents (4 selective versus 6 multi-kinase), and the *a priori* classification of individual drugs is a simplification of continuous kinome-selectivity profiles; in particular, reclassifying lenvatinib (VEGFR-selective but with appreciable FGFR activity) into the multi-kinase group preserved the contrast for ROR (p = 0.033) but reduced it to borderline for IC_025_ (p = 0.058), so the classification of borderline agents materially affects the strength of the contrast. The continuous IC_50_–ROR analysis is correspondingly based on a small sample of 11 VEGFR-TKIs; leave-one-out robustness analysis (Supplementary Table S8) demonstrated that the formal significance of the prespecified cabozantinib-excluded correlation depends on a single pharmacologically discordant data point, and the IC_50_ values themselves are aggregated from heterogeneous published assay systems. Third, the four fruquintinib-PRES cases summarised as focused literature context (Table 1) are restricted to the PubMed-indexed literature and may under-represent atypical PRES presentations (frontal-predominant, brainstem, cerebellar), which are reported in 30%–40% of general PRES cohorts but are less likely to be published as case reports. Finally, we have in this revision applied formal Bonferroni family-wise and Benjamini–Hochberg false-discovery-rate correction to all per-agent tests (Supplementary Table S11) and replaced the ≥3-of-4 screening rule with a principled definition (FDR-significant with positive IC_025_): 13 of 16 evaluable agents are robust signals, and the four selective VEGFR-2 TKIs and bevacizumab survive the most conservative bound by many orders of magnitude. The earlier absence of multiplicity control is therefore resolved.

### 4.9 Future directions and clinical implications

Three priorities follow from these results. First, the tivozanib and ramucirumab signals—the first- and sixth-ranked disproportionality findings in this series and, to our knowledge, under-represented in the dedicated PubMed-indexed case-report literature—warrant independent confirmation in additional pharmacovigilance resources and case-series descriptions. Second, the exploratory IC_50_-based analyses suggest that more refined translational work integrating pharmacology, exposure, and blood-pressure trajectories may help explain inter-agent heterogeneity, but such work must move beyond spontaneous-report data alone. Third, from a clinical standpoint, systematic blood-pressure monitoring and low-threshold neurological evaluation remain prudent for patients receiving selective VEGFR-TKIs, especially during the first treatment weeks or after dose escalation.

These hypothesis-generating findings should be confirmed in designs that overcome the limitations of spontaneous reporting. We propose (i) a prospective cohort of patients initiating selective VEGFR-TKIs, with protocolised blood-pressure monitoring, systemic-exposure (pharmacokinetic) sampling, and standardised neuroimaging endpoints, to establish absolute incidence and exposure–response relationships; and (ii) an indication-stratified comparison (oncology versus IPF/ILD) to test directly whether the nintedanib inverse signal reflects lower vascular selectivity or population/reporting differences. To maximise transparency and limit analytical flexibility, such confirmatory studies should be pre-registered (e.g., on OSF or ClinicalTrials.gov) before data collection, with pre-specified PRES case definitions and analysis plans.

## 5 Conclusion

PRES disproportionality signals varied substantially across the VEGFi/VEGFRi class. Thirteen of the 16 evaluable agents produced robust elevated reporting signals (under formal multiplicity correction), while intravitreal agents showed no positive signal and nintedanib showed an inverse signal likely related to indication channelling. Exploratory IC_50_-based analyses were directionally consistent with a VEGFR-2 selectivity gradient but should be regarded as hypothesis-generating rather than mechanistic proof. Tivozanib and ramucirumab emerged as under-represented signals that warrant independent confirmation, while selective VEGFR-TKI recipients may merit close blood-pressure monitoring and a low threshold for neuroimaging when new neurological symptoms occur. These associations are hypothesis-generating and require confirmation in prospective cohort or registry-based studies.

## Data Availability

The original contributions presented in the study are included in the article/Supplementary Material, further inquiries can be directed to the corresponding author.
