# Supplementary material for: Posterior reversible encephalopathy syndrome across 22 VEGF-pathway inhibitors in the FDA adverse event reporting system: reporting patterns and reporting-interval characteristics
Source: Front Pharmacol. 2026 Jul 9;17:1887454. doi: 10.3389/fphar.2026.1887454 (PMC13392357; doi:10.3389/fphar.2026.1887454)

Supplementary Material

Posterior reversible encephalopathy syndrome across 22 VEGF-pathway inhibitors in the FDA Adverse Event Reporting System: selectivity-stratified reporting patterns and reporting-interval characteristics

**Contents**

• Table S1. Drug synonym dictionary used for FAERS query

• Table S2. MedDRA Preferred Terms used for PRES case ascertainment

• Table S3. Literature concordance of FAERS PRES signals with PubMed-indexed case reports

• Table S4. VEGFR-2 biochemical IC₅₀ values and source references

• Table S5. Sensitivity analyses summary (S1–S4)

• Table S6. STROBE-adapted reporting checklist for FAERS disproportionality study

• Table S7. READUS-PV reporting checklist for disproportionality analysis

• Table S8. IC₅₀-ROR robustness analyses: bootstrap 95% CI and leave-one-out

• Table S9. Sensitivity of the selective-versus-multikinase comparison to lenvatinib classification

• Table S10. Reported indications of PRES-positive reports (openFDA)

• Table S11. Per-agent disproportionality with formal multiplicity correction

• Figure S1. FAERS ROR versus PubMed-indexed case-report volume

• Figure S2. VEGFR-2 biochemical potency versus PRES disproportionality signal (continuous IC₅₀–ROR)

**Supplementary Tables S1-S11 are consolidated in the accompanying Excel workbook (Supplementary_Tables_S1-S11.xlsx). Supplementary Figures S1 and S2 are embedded below.**

**Table S1. Drug synonym dictionary used for FAERS query**

*All synonyms were queried as uppercase exact matches against three FAERS/openFDA fields joined by Boolean OR: patient.drug.openfda.generic_name.exact, patient.drug.openfda.brand_name.exact, and patient.drug.medicinalproduct.exact. Only reports with drugcharacterization = 1 (primary suspect) were included.*

| **Drug (INN)** | **Mechanistic class** | **Brand names** | **Investigational codes** |
| --- | --- | --- | --- |
| Bevacizumab | mAb / decoy (systemic) | Avastin, Mvasi, Zirabev, Alymsys | rhuMAb VEGF |
| Ramucirumab | mAb / decoy (systemic) | Cyramza | IMC-1121B, LY3009806 |
| Aflibercept | mAb / decoy (systemic) | Zaltrap, Eylea | AVE 0005, VEGF-TRAP |
| Ranibizumab | Intravitreal anti-VEGF | Lucentis, Byooviz, Cimerli | rhuFab V2 |
| Brolucizumab | Intravitreal anti-VEGF | Beovu | RTH258, ESBA-1008 |
| Conbercept | Intravitreal anti-VEGF | Lumitin | KH902 |
| Tivozanib | Highly selective VEGFR-TKI | Fotivda | AV-951, KRN951 |
| Axitinib | Highly selective VEGFR-TKI | Inlyta | AG-013736 |
| Fruquintinib | Highly selective VEGFR-TKI | Elunate, Fruzaqla | HMPL-013 |
| Lenvatinib | VEGFR + FGFR selective | Lenvima, Kisplyx | E7080 |
| Vandetanib | VEGFR + RET + EGFR | Caprelsa | ZD6474 |
| Cediranib | VEGFR-TKI (investigational) | Recentin | AZD2171 |
| Brivanib | VEGFR + FGFR (investigational) | — | BMS-582664 |
| Sunitinib | Multi-kinase VEGFR-TKI | Sutent | SU11248 |
| Sorafenib | Multi-kinase VEGFR-TKI | Nexavar | BAY 43-9006 |
| Pazopanib | Multi-kinase VEGFR-TKI | Votrient | GW786034 |
| Cabozantinib | Multi-kinase (MET-primary) | Cabometyx, Cometriq | XL184, BMS-907351 |
| Regorafenib | Multi-kinase VEGFR-TKI | Stivarga | BAY 73-4506 |
| Nintedanib | FGFR-primary + VEGFR + PDGFR | Ofev, Vargatef | BIBF 1120 |
| Apatinib | VEGFR-TKI (China-primary) | Aitan, Rivoceranib | YN968D1 |
| Anlotinib | Multi-kinase (China-primary) | Focus V, Fukewei | AL3818 |
| Surufatinib | VEGFR + FGFR1 + CSF1R | Sulanda | HMPL-012, sulfatinib |

*INN, international nonproprietary name; TKI, tyrosine kinase inhibitor.*

**Table S2. MedDRA Preferred Terms used for PRES case ascertainment**

*PRES ascertainment used a two-tier definition. Narrow (primary analysis) comprised the two exact PRES synonyms; broad (sensitivity analysis S1) added related PTs that may capture atypical presentations.*

| **MedDRA Preferred Term** | **Scope** | **Primary analysis** | **Notes** |
| --- | --- | --- | --- |
| Posterior reversible encephalopathy syndrome | Narrow (primary) | Yes | Canonical PT for PRES |
| Reversible posterior leukoencephalopathy syndrome | Narrow (primary) | Yes | Historical synonym |
| Hypertensive encephalopathy | Broad | Sensitivity only | Captures PRES coded under HTN umbrella |
| Leukoencephalopathy | Broad | Sensitivity only | Captures atypical PRES presentations |
| Toxic leukoencephalopathy | Broad | Sensitivity only | Drug-associated leukoencephalopathy |
| Cortical blindness | Excluded | No | Non-specific for PRES |

*MedDRA version 25.0 or later. PT, Preferred Term.*

**Table S3. Literature concordance of FAERS PRES signals with PubMed-indexed case reports**

Targeted PubMed search executed 20 April 2026; English-language PubMed-indexed case reports, 2004–2026. Case counts are reported as exact numbers for sparse literatures and as conservative lower-bound thresholds for better-documented literatures. Conference abstracts and non-English literature were recorded but not included in the primary count.

| **Drug** | **Class** | **FAERS ROR (95% CI)** | **PubMed cases (n)** | **First year** | **Key references** | **Note** |
| --- | --- | --- | --- | --- | --- | --- |
| Tivozanib | TKI selective | 16.68 (9.45–29.45) | 0 | — | 1 non-indexed conference abstract (SHM 2022) | Novel signal |
| Lenvatinib | TKI selective | 11.62 (9.98–13.52) | ≥5 | 2018 | Chae 2018; Tseng 2022; LEAP combo 2023 | Well-documented |
| Fruquintinib | TKI selective | 11.38 (7.06–18.34) | 4 | 2023 | Wang 2023; Ledet 2025; ElManfalouty 2025; Ioannidis 2025 | mCRC predominant |
| Axitinib | TKI selective | 9.74 (7.90–12.01) | 1–2 | 2017 | Nakamura 2017 | Under-reported |
| Bevacizumab | mAb | 9.29 (8.51–10.14) | ≥20 | 2006 | Glusker 2006; Seet 2012 review (n=12); many others | Most prevalent in literature |
| Ramucirumab | mAb | 7.56 (4.97–11.49) | 0–1 | — | No dedicated PubMed case report identified | Novel signal |
| Pazopanib | TKI multi-kinase | 6.05 (4.84–7.56) | ≥6 | 2012 | Chelis 2012; Deguchi 2018; Savaliya 2023 | Well-documented |
| Vandetanib | Multi-kinase | 5.87 (2.44–14.14) | 2 | 2016 | Kamiya-Matsuoka 2016 (2 DIPG) | Pediatric |
| Regorafenib | TKI multi-kinase | 4.90 (3.36–7.15) | 1 | 2014 | Myint 2014 | Under-reported |
| Sunitinib | TKI multi-kinase | 3.49 (2.76–4.41) | ≥8 | 2007 | Martín 2007; Padhy 2011; Costa 2014; others | Well-documented |
| Sorafenib | TKI multi-kinase | 2.71 (1.86–3.95) | 3–4 | 2010 | Govindarajan 2010; Ohba 2016; pediatric 2016 | Well-documented |
| Cabozantinib | Multi-kinase (MET) | 2.00 (1.47–2.70) | 1 | 2020 | Reddy 2020 (first reported) | Under-reported |
| Aflibercept | Decoy | 1.98 (1.38–2.83) | 1 | 2011 | Tlemsani 2011 review | Literature-scarce |
| Nintedanib | Inverse | 0.34 (0.14–0.82) | 0 | — | No PubMed case report identified | Inverse signal |

*CI, confidence interval; DIPG, diffuse intrinsic pontine glioma; mAb, monoclonal antibody; mCRC, metastatic colorectal cancer; MET, mesenchymal-epithelial transition receptor; ROR, reporting odds ratio; TKI, tyrosine kinase inhibitor.*

**Table S4. VEGFR-2 biochemical IC₅₀ values and source references**

Recombinant VEGFR-2 (KDR) kinase enzymatic IC₅₀ or Ki from the original drug-discovery manuscript. Assay conditions are not harmonized across sources; values are therefore used only as ordinal selectivity markers in an exploratory, hypothesis-generating analysis.

| **Drug** | **Mechanistic class** | **VEGFR-2 IC₅₀ (nM)** | **Source reference** | **Sensitivity exclusion** |
| --- | --- | --- | --- | --- |
| Cabozantinib | Multi-kinase (MET-primary) | 0.035 | Yakes 2011 Mol Cancer Ther | Sensitivity A |
| Tivozanib | Highly selective VEGFR | 0.21 | Nakamura 2006 Cancer Res |  |
| Axitinib | Highly selective VEGFR | 0.25 | Hu-Lowe 2008 Clin Cancer Res |  |
| Fruquintinib | Highly selective VEGFR | 2.0 | Sun 2014 Cancer Biol Ther |  |
| Lenvatinib | VEGFR + FGFR | 4.0 | Matsui 2008 Clin Cancer Res |  |
| Regorafenib | Multi-kinase | 4.2 | Wilhelm 2011 Int J Cancer |  |
| Nintedanib | FGFR-primary | 21.0 | Hilberg 2008 Cancer Res | Sensitivity B |
| Pazopanib | Multi-kinase | 30.0 | Kumar 2007 Mol Cancer Ther |  |
| Vandetanib | VEGFR + RET + EGFR | 40.0 | Hennequin 2002 J Med Chem |  |
| Sunitinib | Multi-kinase | 80.0 | Mendel 2003 Clin Cancer Res |  |
| Sorafenib | Multi-kinase | 90.0 | Wilhelm 2004 Cancer Res |  |

Exploratory Spearman rank correlation with log₁₀ ROR: Primary (all 11): ρ = −0.38, p = 0.25. Sensitivity A (excluding cabozantinib, n = 10): ρ = −0.77, p = 0.009. Sensitivity B (excluding cabozantinib and nintedanib, n = 9): ρ = −0.88, p = 0.002.

**Table S5. Sensitivity analyses summary**

Summary of the four tabulated sensitivity analyses (S1-S4): broad PT definition, physician-reporter only, post-2014 window, and within-class comparator. Leave-one-drug-out and pooled selective-versus-multi-kinase contrasts are reported in the main manuscript Results §3.4. The full table is provided below and, as a machine-readable copy, in the accompanying Excel workbook (sheet S5).

| **Sensitivity analysis** | **Definition** | **PRES report count** | **Top-5 rank stability** | **Key observation** |
| --- | --- | --- | --- | --- |
| S1 Broad PT definition | PRES preferred terms plus 3 low-specificity broad terms (hypertensive encephalopathy, leukoencephalopathy, toxic leukoencephalopathy) | ~11,600 (~20% more than primary) | Unchanged (all 5 top drugs retained rank) | Broad PTs increased case capture without materially altering top-rank ordering |
| S2 Physician-reporter only | Reports with primarysource.qualification ∈ {1, 2, 3} (physician / pharmacist / other-HCP) | 6,821 | Unchanged; tivozanib retains rank 1; fruquintinib and lenvatinib retain top-3 | Consumer-only reports did not drive the signals |
| S3 Post-2014 window | Receipt dates Q1 2014 onwards | 9,014 | Unchanged | No material shift from earliest quarters |
| S4 Within-class comparator | Denominator restricted to 22-drug VEGFi/VEGFRi subset only | 9,745 (same numerator) | Selectivity gradient preserved; inverse nintedanib signal retained | Within-class comparison reproduces the primary selectivity ranking |

*All sensitivity analyses used the same PRES numerator and disproportionality method as the primary analysis; see Methods §2.6 for definitions. Rank stability refers to whether the top-5 drugs by ROR remained in the same order as the primary analysis.*

**Table S6. STROBE-adapted reporting checklist for FAERS disproportionality study**

Completed STROBE-adapted reporting checklist, mapping each recommended item to its location in the manuscript. The full checklist is provided below and, as a machine-readable copy, in the accompanying Excel workbook (sheet S6).

| **Section/Item** | **#** | **STROBE Recommendation** | **Adaptation for FAERS Disproportionality Study** | **Reported in Manuscript** | **Page / Section** |
| --- | --- | --- | --- | --- | --- |
| **Title and abstract** |  |  |  |  |  |
| Title and abstract | 1(a) | Indicate the study's design with a commonly used term in the title or the abstract | FAERS disproportionality study explicitly identified in title ("FDA Adverse Event Reporting System analysis") and abstract Methods | Yes | Title; Abstract Methods |
| Title and abstract | 1(b) | Provide in the abstract an informative and balanced summary of what was done and what was found | Structured abstract: Objectives, Methods, Results, Conclusion | Yes | Abstract (§Abstract) |
| **Introduction** |  |  |  |  |  |
| Background/rationale | 2 | Explain the scientific background and rationale for the investigation being reported | Class-level VEGFi/VEGFRi-PRES association established; within-class gradient not previously tested | Yes | Introduction §1 |
| Objectives | 3 | State specific objectives, including any prespecified hypotheses | Four prespecified hypotheses (H1-H4) stated in Introduction | Yes | Introduction §1, final paragraph |
| **Methods** |  |  |  |  |  |
| Study design | 4 | Present key elements of study design early in the paper | Observational pharmacovigilance disproportionality analysis of spontaneous reporting database | Yes | Methods §2.1; Abstract Methods |
| Setting | 5 | Describe the setting, locations, and relevant dates | FAERS via openFDA API; 2010-Q1 to 2026-Q1; global spontaneous reporting | Yes | Methods §2.1 |
| Participants | 6 | Give the eligibility criteria, and the sources and methods of selection of participants | ICSRs as units of analysis; inclusion = primary suspect (drugcharacterization=1); 22-drug exact-match query against three openFDA fields (generic_name.exact, brand_name.exact, medicinalproduct.exact) | Yes | Methods §2.1-2.2; Supp Table S1 |
| Variables | 7 | Clearly define all outcomes, exposures, predictors, potential confounders, and effect modifiers | Outcome: PRES (MedDRA PT-level narrow definition; broad in sensitivity); Exposure: 22 VEGFi/VEGFRi agents; effect modifiers: route, indication | Yes | Methods §2.3; Supp Table S2 |
| Data sources/measurement | 8 | For each variable of interest, give sources of data and details of methods of assessment | FAERS publicly accessible via openFDA API; MedDRA PT-coded events; ROR/PRR/IC025/EBGM computed from 2×2 tables | Yes | Methods §2.1, §2.4 |
| Bias | 9 | Describe any efforts to address potential sources of bias | Notoriety/Weber effects, indication channelling, under-reporting, MedDRA misclassification, primary-suspect restriction trade-off, geographic FAERS coverage explicitly discussed in Limitations | Yes | Discussion §4.8 (Limitations) |
| Study size | 10 | Explain how the study size was arrived at | All FAERS reports in the query window analyzed; no sampling. 19,980,897 primary-suspect reports; 9,745 PRES PTs | Yes | Results §3.1 |
| Quantitative variables | 11 | Explain how quantitative variables were handled in the analyses | Disproportionality metrics computed at drug level; reporting interval analyzed as continuous (Weibull, exploratory) | Yes | Methods §2.4-2.5 |
| Statistical methods | 12(a) | Describe all statistical methods, including those used to control for confounding | Four disproportionality methods (ROR, PRR, BCPNN-IC with IC025, EBGM); robustness criterion (screening ≥3 of 4; in revision confirmed by Bonferroni and Benjamini-Hochberg FDR correction with IC025>0, Supp Table S11); robustness across pre-specified sensitivity analyses (S1-S6) | Yes | Methods §2.4, §2.6 |
| Statistical methods | 12(b) | Describe any methods used to examine subgroups and interactions | Sex-stratified; physician-only sensitivity; post-2014 sensitivity; within-class comparator sensitivity; leave-one-out; pooled subclass | Yes | Methods §2.6; Supp Table S5 |
| Statistical methods | 12(c) | Explain how missing data were addressed | Only reports with both drugstartdate and receivedate populated and internally consistent retained for reporting-interval analysis (Weibull); missing demographics handled through complete-case | Yes | Methods §2.5 |
| Statistical methods | 12(d) | If applicable, describe analytical methods taking account of sampling strategy | Not applicable; full FAERS query window analyzed | N/A | — |
| Statistical methods | 12(e) | Describe any sensitivity analyses | Six prespecified sensitivity analyses (S1 broad PT; S2 physician-only; S3 post-2014; S4 within-class denominator; S5 leave-one-out; S6 pooled subclass) | Yes | Methods §2.6; Supp Table S5 |
| **Results** |  |  |  |  |  |
| Participants | 13 | Report numbers of individuals at each stage of study | 19,980,897 primary-suspect reports; 420,386 VEGFi-class; 1,128 VEGFi-PRES; 9,745 total PRES across FAERS | Yes | Results §3.1 |
| Descriptive data | 14(a) | Give characteristics of study participants | Primary-suspect report volumes and PRES counts by drug (Table 1) | Yes | Results §3.1; Table 1 |
| Outcome data | 15 | Report numbers of outcome events or summary measures over time | PRES report counts and disproportionality metrics for all 22 agents (Table 2) | Yes | Results §3.2; Table 2 |
| Main results | 16(a) | Give unadjusted estimates and, if applicable, confounder-adjusted estimates and their precision | ROR with 95% CI, PRR, IC025, EBGM reported per drug; signal robustness flagged | Yes | Table 2 |
| Main results | 16(b) | Report category boundaries when continuous variables were categorized | Mechanistic subclasses (mAb/decoy, intravitreal, selective VEGFR-TKI, multi-kinase, FGFR-primary) defined a priori per pharmacological characteristics | Yes | Methods §2.2, §2.7; Table 1 |
| Main results | 16(c) | If relevant, consider translating estimates of relative risk into absolute risk | Not applicable; disproportionality reflects reporting signal not absolute risk. Explicit interpretive caveat provided | Yes | Discussion §4.8 |
| Other analyses | 17 | Report other analyses done (e.g. subgroup, sensitivity, interactions) | Exploratory IC50-ROR correlation; reporting-interval Weibull (exploratory); literature concordance scan; all six prespecified sensitivity analyses | Yes | Results §3.4-3.8; Supp Table S5 |
| **Discussion** |  |  |  |  |  |
| Key results | 18 | Summarise key results with reference to study objectives | 13/22 agents with robust signals (FDR-corrected); selectivity gradient directionally consistent (exploratory); intravitreal null; nintedanib inverse; tivozanib/ramucirumab under-represented in literature | Yes | Discussion §4.1 |
| Limitations | 19 | Discuss limitations, including sources of potential bias or imprecision | Notoriety/Weber, indication channelling, under-reporting, geographic coverage gaps, misclassification, confounding by HTN/CKD/concomitants, small case counts (tivozanib n=12, nintedanib n=5), reporting interval ≠ true onset, IC50 heterogeneity across assay sources | Yes | Discussion §4.8 |
| Interpretation | 20 | Give a cautious overall interpretation considering objectives, limitations, multiplicity of analyses, results from similar studies, and other relevant evidence | All findings framed as reporting signals rather than risk; IC50-ROR explicitly exploratory; Weibull explicitly reporting-interval distribution; replication priorities identified | Yes | Discussion §4.1-4.7, §4.9 |
| Generalisability | 21 | Discuss the generalisability of the study results | FAERS coverage limitations (geographic, specialty) and reporter behaviour discussed; recommendations conditioned accordingly | Yes | Discussion §4.8-4.9 |
| **Other information** |  |  |  |  |  |
| Funding | 22 | Give the source of funding and the role of the funders for the present study and, if applicable, for the original study on which the present article is based | No specific funding; statement included in Declarations | Yes | Declarations |

*Notes: This checklist is adapted from STROBE for observational studies (von Elm 2007 Lancet 370:1453) and aligned with READUS-PV reporting principles for disproportionality analyses of individual case safety reports (Fusaroli, Salvo, Khouri, Raschi et al. 2024 Drug Safety 47:585-599). Items marked "N/A" are not applicable to spontaneous-report disproportionality designs.*

**Table S7. READUS-PV reporting checklist for disproportionality analysis**

Completed READUS-PV reporting checklist for disproportionality analyses of individual case safety reports. The full checklist is provided below and, as a machine-readable copy, in the accompanying Excel workbook (sheet S7).

| **Section/Item** | **#** | **READUS-PV Recommendation** | **Reported in Manuscript** | **Page / Section** |
| --- | --- | --- | --- | --- |
| **Title and abstract** |  |  |  |  |
| Title | 1.1 | Identify the study as a disproportionality analysis using ICSR data in the title | Title contains "FDA Adverse Event Reporting System analysis" identifying the data source and study design | Title |
| Abstract | 1.2 | Provide a structured abstract including objectives, methods (data source, study population, statistical methods), key results, and conclusions | Abstract structured under Objectives, Methods, Results, Conclusion; FAERS source, 22-drug class, 4 disproportionality methods all specified | Abstract |
| **Introduction** |  |  |  |  |
| Background and rationale | 2.1 | Explain the scientific background, including the drug(s), the adverse event(s), and the rationale for the disproportionality analysis | Class-level VEGF-pathway / PRES association established; within-class gradient untested previously | Introduction §1 |
| Objectives | 2.2 | State specific, prespecified objectives and, if appropriate, hypotheses | Four prespecified hypotheses (H1–H4) presented in Introduction final paragraph | Introduction §1 |
| **Methods** |  |  |  |  |
| Data source | 3.1 | Describe the data source, including time frame, version, and any preprocessing or curation | FAERS via openFDA API; 2010-Q1 to 2026-Q1; duplicate handling described; query strategy specified | Methods §2.1 |
| Study population | 3.2 | Define the study population (the units of analysis: reports, drug-report pairs, etc.) and the inclusion/exclusion criteria | Units of analysis: ICSRs as primary suspect (drugcharacterization=1); 22-drug exact-match query; sensitivity restrictions described | Methods §2.1-2.2 |
| Drug exposure ascertainment | 3.3 | Specify how drugs are identified, including synonyms, brand/generic names, ATC codes, and handling of free-text | Up to 6 synonyms per drug (INN, brand names, investigational codes) queried against three openFDA fields by Boolean OR; uppercase exact match; full dictionary in Supp Table S1 | Methods §2.2; Supp Table S1 |
| Event ascertainment | 3.4 | Specify the source dictionary (e.g., MedDRA) and version, the terms used for event identification, and the level of the hierarchy | MedDRA PT-level; narrow definition (2 PTs); broad definition (5 PTs) for sensitivity only | Methods §2.3; Supp Table S2 |
| Disproportionality measures | 3.5 | Specify the disproportionality method(s) used, with explicit formulae, thresholds, and rationale for choice | Four methods reported: ROR, PRR, BCPNN-IC with IC025, EBGM; explicit 2x2 table formulae; thresholds (ROR lower CI >1, a ≥3; PRR ≥2 with chi-sq ≥4; IC025 >0; EBGM ≥2); robust signal = ≥3 of 4 positive | Methods §2.4 |
| Confounding and biases | 3.6 | Describe how confounding by indication, by co-medication, and other reporting biases were addressed | Notoriety/Weber effects, indication channelling, primary-suspect restriction trade-off, MedDRA misclassification, geographic FAERS coverage limitations all addressed explicitly in Discussion §4.8 | Methods §2.6 (sensitivity analyses); Discussion §4.8 |
| Subgroup and sensitivity analyses | 3.7 | Pre-specify any subgroup, sensitivity, or additional analyses | 6 prespecified sensitivity analyses (S1-S6) defined a priori; tabulated subset (S1-S4) in Supp Table S5 | Methods §2.6; Supp Table S5 |
| **Results** |  |  |  |  |
| Descriptive statistics | 4.1 | Report total number of reports, distribution of reports by drug/event, demographic distribution where relevant | 19,980,897 primary-suspect reports; 9,745 PRES PT reports across FAERS; 420,386 VEGFi-class; 1,128 VEGFi-PRES; per-drug counts in Table 1 | Results §3.1; Table 1 |
| Disproportionality results | 4.2 | Report disproportionality estimates with measures of uncertainty (95% CI or credibility interval); state robust signal classification | ROR with 95% CI, PRR with chi-square, IC025, EBGM all reported per drug in Table 2; robust signal flagged | Results §3.2; Table 2 |
| Sensitivity results | 4.3 | Report the results of prespecified sensitivity analyses, including direction and magnitude of change | All 6 sensitivity analyses reported; rank stability of top-5 drugs preserved across S1-S4; tabulated in Supp Table S5 | Results §3.7; Supp Table S5 |
| Time-to-event-like analyses | 4.4 | If a time-to-onset analysis is reported, clarify whether it reflects true onset or reporting delay; report descriptors with caveats | Weibull modelling explicitly described as reporting-delay distribution (NOT clinical hazard or time-to-event); shape parameter interpreted as descriptor only | Methods §2.5; Results §3.5; Discussion §4.5 |
| **Discussion** |  |  |  |  |
| Interpretation | 5.1 | Interpret findings cautiously as reporting signals, NOT as risk estimates or causal associations; consider biological plausibility, prior evidence, and study limitations | All findings consistently framed as "disproportionality signal" / "reporting signal"; word "risk" appears only once and in a strict narrative context; IC50-ROR explicitly labelled exploratory and hypothesis-generating | Discussion §4.1, §4.2, §4.8 |
| Limitations | 5.2 | Explicitly discuss limitations of spontaneous reporting data, including underreporting, channelling, notoriety/Weber, missing denominators, and event misclassification | Dedicated Limitations subsection covering notoriety/Weber, indication channelling, under-reporting, denominator absence, MedDRA misclassification, small-n agents (tivozanib n=12, nintedanib n=5), geographic coverage gaps (anlotinib in Chinese market) | Discussion §4.8 |
| Generalizability | 5.3 | Discuss the generalizability of findings beyond the specific reporting system and time window | FAERS coverage limitations explicit (geography, specialty, reporter behaviour); recommendations conditioned on these | Discussion §4.8-4.9 |
| **Other information** |  |  |  |  |
| Funding and conflicts of interest | 6.1 | Report funding sources and conflicts of interest | No specific funding stated; no competing interests declared | Declarations |
| Data sharing | 6.2 | Describe accessibility of raw data, aggregated data, and analytic code | FAERS raw data publicly available via openFDA API; aggregated 2x2 tables provided in Supplementary; analytic code available on reasonable request and to be deposited at persistent DOI on acceptance | Declarations; Methods §2.9 |

*Reference: Fusaroli M, Salvo F, Bergvall T, Bate A, Carnovale C, Cracowski JL, Khouri C, Raschi E, et al. The REporting of A Disproportionality analysis for drUg Safety signal detection using individual case safety reports in PharmacoVigilance (READUS-PV): explanation and elaboration. Drug Safety 2024;47:585–599. doi:10.1007/s40264-024-01423-7*

**Table S8. IC₅₀-ROR robustness analyses: bootstrap 95% CI and leave-one-out**

Bootstrap 95% confidence intervals and drug-level leave-one-out for the exploratory log₁₀ VEGFR-2 IC₅₀ versus log₁₀ ROR Spearman correlation. The full data are provided below and, as a machine-readable copy, in the accompanying Excel workbook (sheet S8).

**Panel A. Spearman ρ with bootstrap 95% CI by prespecified exclusion**

| **Analysis** | **n** | **Spearman ρ** | **p-value** | **Bootstrap 95% CI** |
| --- | --- | --- | --- | --- |
| Primary (all 11 VEGFR-TKIs) | 11 | -0.382 | 0.247 | [-0.93, +0.32] |
| Sensitivity A: exclude Cabozantinib (MET-primary per FDA label) | 10 | -0.770 | 0.009 | [-0.99, -0.24] |
| Sensitivity B: exclude Cabozantinib + Nintedanib (FGFR-primary, non-oncology) | 9 | -0.883 | 0.002 | [-1.00, -0.47] |

**Panel B. Drug-level leave-one-out (LOO) analysis: each of 11 drugs iteratively removed; ρ recomputed on remaining 10**

| **Drug dropped** | **VEGFR-2 IC50 (nM)** | **PRES ROR** | **Spearman ρ on remaining 10** | **p-value** |
| --- | --- | --- | --- | --- |
| Cabozantinib | 0.035 | 2.00 | -0.770 | 0.0092 |
| Tivozanib | 0.21 | 16.68 | -0.285 | 0.4250 |
| Axitinib | 0.25 | 9.74 | -0.321 | 0.3655 |
| Fruquintinib | 2 | 11.38 | -0.321 | 0.3655 |
| Lenvatinib | 4 | 11.62 | -0.321 | 0.3655 |
| Regorafenib | 4.2 | 4.90 | -0.345 | 0.3282 |
| Nintedanib | 21 | 0.34 | -0.370 | 0.2931 |
| Pazopanib | 30 | 6.05 | -0.382 | 0.2763 |
| Vandetanib | 40 | 5.87 | -0.382 | 0.2763 |
| Sunitinib | 80 | 3.49 | -0.345 | 0.3282 |
| Sorafenib | 90 | 2.71 | -0.345 | 0.3282 |

*Summary: 1 of 11 leave-one-out iterations yielded p < 0.05; significance preserved only when cabozantinib was removed. This demonstrates that the prespecified cabozantinib-excluded correlation is exclusion-sensitive and should be interpreted as exploratory and hypothesis-generating.*

**Table S9. Sensitivity of the selective-versus-multikinase comparison to the classification of lenvatinib**

*ROR, reporting odds ratio; MWU, Mann–Whitney U test (one-sided, selective > multikinase). The higher PRES reporting of selective versus multikinase VEGFR-TKIs is statistically significant under all three classification choices (p = 0.005–0.033). Complete rank separation holds when lenvatinib is grouped as selective (primary analysis) or excluded, but is lost when lenvatinib is reclassified as multikinase, because its own reporting signal (ROR ≈ 11.4) then becomes the highest value within the multikinase group. The full data are provided below and, as a machine-readable copy, in the accompanying Excel workbook (sheet S9).*

| **Scenario** | **Selective (n; median ROR)** | **Multikinase (n; median ROR)** | **Complete rank separation** | **MWU one-sided p** |
| --- | --- | --- | --- | --- |
| A. Lenvatinib = selective (primary) | 4; 11.50 | 6; 4.19 | Yes | 0.0048 |
| B. Lenvatinib reclassified as multikinase | 3; 11.38 | 7; 4.90 | No | 0.0333 |
| C. Lenvatinib excluded | 3; 11.38 | 6; 4.19 | Yes | 0.0119 |

*ROR, reporting odds ratio; MWU, Mann–Whitney U test (one-sided, selective > multikinase). The higher PRES reporting of selective versus multikinase VEGFR-TKIs is statistically significant under all three classification choices (p = 0.005–0.033). Complete rank separation holds when lenvatinib is grouped as selective (primary analysis) or excluded, but is lost when lenvatinib is reclassified as multikinase, because its own reporting signal (ROR ≈ 11.4) then becomes the highest value within the multikinase group.*

**Table S10. Reported indications of PRES-positive reports for representative agents (openFDA)**

*Indication-string frequencies among PRES-positive reports (openFDA drugindication); a report may list more than one indication, and the field reflects all drugs on a report. No denominators — descriptive context only, not a rate comparison. Every nintedanib PRES report carried a non-oncological fibrotic indication and none arose in oncology, whereas the selective VEGFR-TKI reports were uniformly oncological. The small nintedanib count (n = 5) precludes any formal matched comparison. The full data are provided below and, as a machine-readable copy, in the accompanying Excel workbook (sheet S10).*

| **Agent** | **Reported indications among PRES-positive reports (n)** | **Population** |
| --- | --- | --- |
| Nintedanib | Idiopathic pulmonary fibrosis (3); interstitial lung disease (2); systemic sclerosis (2) | Non-oncology / fibrotic only |
| Fruquintinib | Metastatic colorectal / colon / rectal cancer (≈11) | Oncology |
| Lenvatinib | Endometrial/uterine (≈30); renal cell (≈25); thyroid (≈9); hepatocellular (≈5) | Oncology |
| Axitinib | Metastatic / renal cell carcinoma (≈14) | Oncology |
| Tivozanib | Renal cell carcinoma (2) | Oncology |

*Indication-string frequencies among PRES-positive reports (openFDA drugindication); a report may list more than one indication, and the field reflects all drugs on a report. No denominators — descriptive context only, not a rate comparison. Every nintedanib PRES report carried a non-oncological fibrotic indication and none arose in oncology, whereas the selective VEGFR-TKI reports were uniformly oncological. The small nintedanib count (n = 5) precludes any formal matched comparison.*

**Table S11. Per-agent disproportionality with formal multiplicity correction**

Two-sided Fisher exact test per drug–PRES 2×2 table for the 16 VEGFi/VEGFRi agents with at least one primary-suspect PRES report (a ≥ 1), with Bonferroni family-wise correction (m = 16; a conservative literal bound at α = 0.05/88 is also shown) and Benjamini–Hochberg false-discovery-rate correction. A robust elevated signal is defined as one that survives BH-FDR at q < 0.05 and has a positive Bayesian IC025. Agents are ordered by ROR.

| **Drug** | **n** | **ROR (95% CI)** | **IC025** | **Fisher exact p** | **Bonferroni p (m=16)** | **BH-FDR q** | **Survives α=0.05/88** | **Robust (FDR & IC025>0)** |
| --- | --- | --- | --- | --- | --- | --- | --- | --- |
| Tivozanib | 12 | 16.68 (9.45–29.45) | 3.23 | 2.2×10⁻¹¹ | 3.5×10⁻¹⁰ | 4.4×10⁻¹¹ | Yes | Yes |
| Lenvatinib | 170 | 11.62 (9.98–13.52) | 3.29 | 4.6×10⁻¹¹⁵ | 7.4×10⁻¹¹⁴ | 3.7×10⁻¹¹⁴ | Yes | Yes |
| Fruquintinib | 17 | 11.38 (7.06–18.34) | 2.82 | 6.7×10⁻¹³ | 1.1×10⁻¹¹ | 1.8×10⁻¹² | Yes | Yes |
| Axitinib | 89 | 9.74 (7.90–12.01) | 2.97 | 3.8×10⁻⁵⁵ | 6.1×10⁻⁵⁴ | 2.0×10⁻⁵⁴ | Yes | Yes |
| Bevacizumab | 532 | 9.29 (8.51–10.14) | 3.02 | <1×10⁻³⁰⁰ | <1×10⁻³⁰⁰ | <1×10⁻³⁰⁰ | Yes | Yes |
| Ramucirumab | 22 | 7.56 (4.97–11.49) | 2.31 | 9.5×10⁻¹³ | 1.5×10⁻¹¹ | 2.2×10⁻¹² | Yes | Yes |
| Apatinib | 1 | 6.46 (0.91–46.05) | -0.13 | 0.1437 | 1 | 0.1437 | No | No |
| Pazopanib | 78 | 6.05 (4.84–7.56) | 2.27 | 1.5×10⁻³⁴ | 2.4×10⁻³³ | 6.0×10⁻³⁴ | Yes | Yes |
| Vandetanib | 5 | 5.88 (2.44–14.13) | 1.29 | 0.0019 | 0.0297 | 0.0021 | No | Yes |
| Regorafenib | 27 | 4.90 (3.35–7.15) | 1.74 | 5.0×10⁻¹¹ | 8.0×10⁻¹⁰ | 8.9×10⁻¹¹ | Yes | Yes |
| Sunitinib | 70 | 3.49 (2.76–4.41) | 1.46 | 4.0×10⁻¹⁸ | 6.4×10⁻¹⁷ | 1.3×10⁻¹⁷ | Yes | Yes |
| Sorafenib | 27 | 2.71 (1.86–3.95) | 0.89 | 6.2×10⁻⁶ | 9.9×10⁻⁵ | 9.9×10⁻⁶ | Yes | Yes |
| Cabozantinib | 42 | 2.00 (1.47–2.70) | 0.56 | 4.5×10⁻⁵ | 7.2×10⁻⁴ | 6.5×10⁻⁵ | Yes | Yes |
| Aflibercept | 30 | 1.98 (1.38–2.83) | 0.47 | 6.8×10⁻⁴ | 0.0109 | 8.4×10⁻⁴ | No | Yes |
| Nintedanib | 5 | 0.34 (0.14–0.82) | -2.82 | 0.0082 | 0.1306 | 0.0087 | No | No (inverse) |
| Ranibizumab | 1 | 0.08 (0.01–0.56) | -6.50 | 6.6×10⁻⁵ | 0.0011 | 8.8×10⁻⁵ | Yes | No (inverse) |

*ROR, reporting odds ratio; IC025, lower 95% credibility bound of the Bayesian information component (BCPNN). Fisher exact p two-sided; Bonferroni p (m=16) = Fisher p × 16, capped at 1. “Survives α=0.05/88” denotes the conservative literal Bonferroni bound (Fisher p < 0.05/88). Nintedanib and ranibizumab carry inverse signals and are not robust elevated signals by definition; apatinib (n=1) survives no correction. Full data also provided in the accompanying Excel workbook (sheet S11); reproducible from multiplicity_correction_R6.R.*

**Figure S1. FAERS ROR versus PubMed-indexed case-report volume**


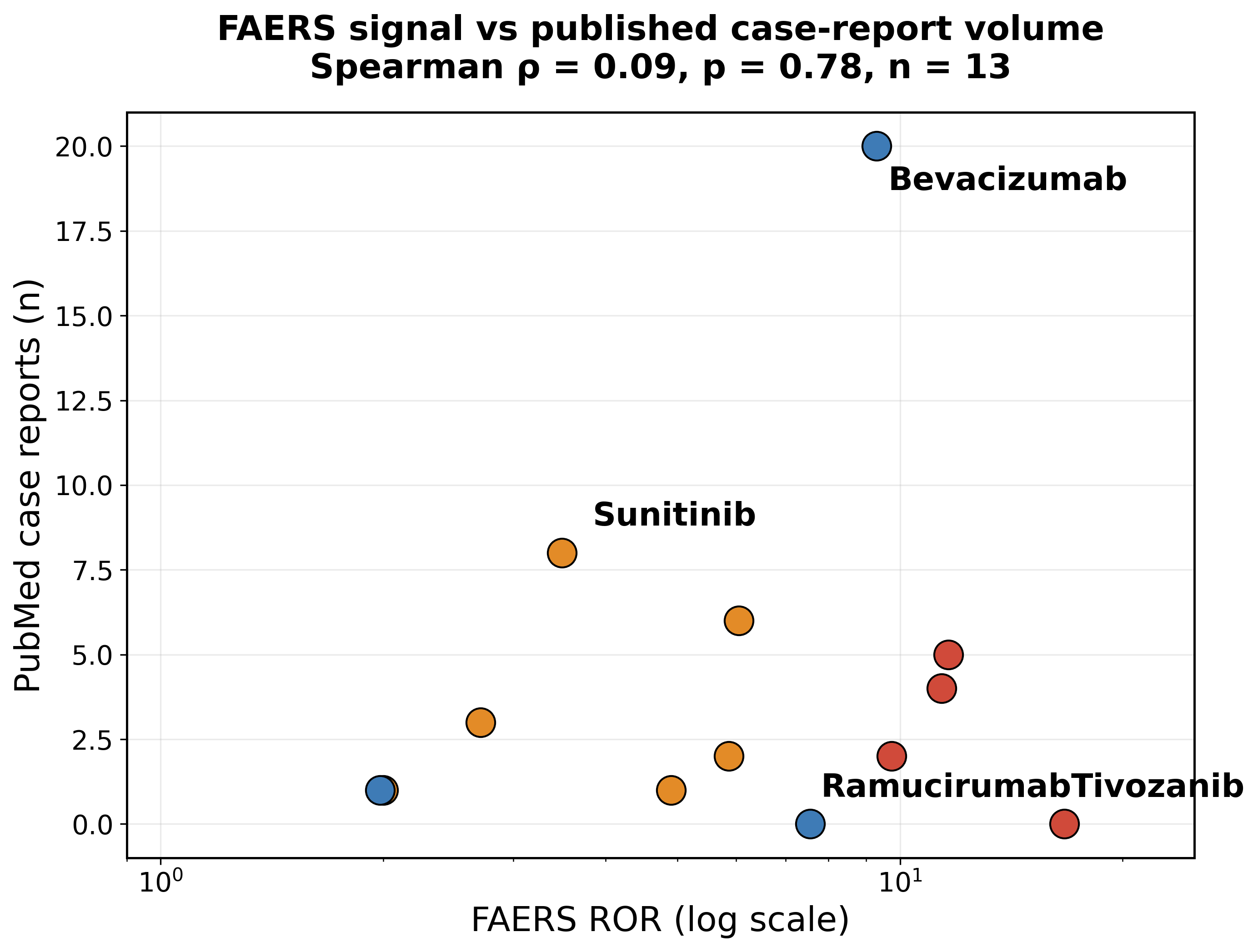


Scatter plot of FAERS ROR (x, log scale) versus the number of PubMed-indexed published PRES case reports (y) across the 13 VEGFi/VEGFRi agents with computable ROR. Spearman’s rank correlation ρ = 0.09, p = 0.78. The absence of concordance highlights the non-equivalence of spontaneous-report disproportionality and case-report volume.

**Figure S2. VEGFR-2 biochemical potency versus PRES disproportionality signal (continuous IC₅₀–ROR)**

*Scatter plot of log₁₀ VEGFR-2 IC₅₀ (nM) against log₁₀ ROR for the 11 VEGFR-TKIs with available biochemical IC₅₀ (Table S4). Relocated from the main text (former Figure 3) at first revision; presented as exploratory and hypothesis-generating only and does not inform the clinical interpretation. Spearman ρ: primary (all 11) = −0.38, p = 0.25; prespecified sensitivity excluding cabozantinib = −0.77, p = 0.009 (n = 10). Figure image shown below.*


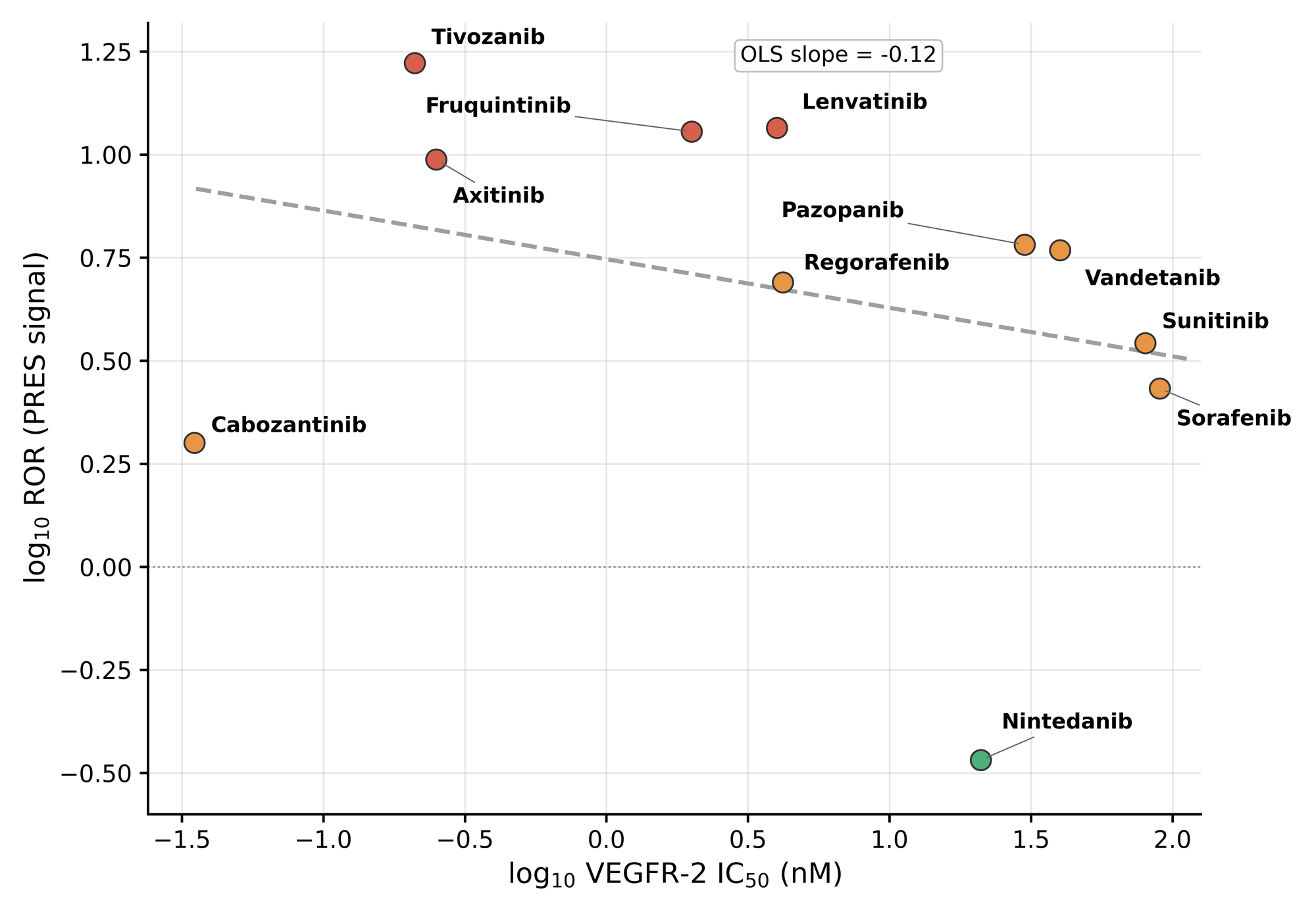

Supplement: Supplementary file 1 [file Supplementaryfile1.docx]
